# Deoxyguanosine kinase deficiency: natural history and liver transplant outcome

**DOI:** 10.1093/braincomms/fcae160

**Published:** 2024-05-06

**Authors:** Eleonora Manzoni, Sara Carli, Pauline Gaignard, Lea Dewi Schlieben, Michio Hirano, Dario Ronchi, Emmanuel Gonzales, Masaru Shimura, Kei Murayama, Yasushi Okazaki, Ivo Barić, Danijela Petkovic Ramadza, Daniela Karall, Johannes Mayr, Diego Martinelli, Chiara La Morgia, Guido Primiano, René Santer, Serenella Servidei, Céline Bris, Aline Cano, Francesca Furlan, Serena Gasperini, Nolwenn Laborde, Costanza Lamperti, Dominic Lenz, Michelangelo Mancuso, Vincenzo Montano, Francesca Menni, Olimpia Musumeci, Victoria Nesbitt, Elena Procopio, Cécile Rouzier, Christian Staufner, Jan-Willem Taanman, Galit Tal, Chiara Ticci, Duccio Maria Cordelli, Valerio Carelli, Vincent Procaccio, Holger Prokisch, Caterina Garone

**Affiliations:** Department of Medical and Surgical Sciences, Alma Mater Studiorum University of Bologna, Bologna 40138, Italy; IRCCS Istituto delle Scienze Neurologiche, UO Neuropsichiatria dell’età Pediatrica di Bologna, Bologna 40124, Italy; Department of Medical and Surgical Sciences, Alma Mater Studiorum University of Bologna, Bologna 40138, Italy; Department of Biochemistry, Bicêtre Hospital, Reference Center for Mitochondrial Disease, University of Paris-Saclay, Assistance Publique-Hôpitaux de Paris, Paris 94275, France; School of Medicine, Institute of Human Genetics, Technical University of Munich, Munich, 80333 Germany; Institute of Neurogenomics, Computational Health Center, Helmholtz Zentrum München, Neuherberg 80333, Germany; H. Houston Merritt Neuromuscular Research Center, Department of Neurology, Columbia University Irving Medical Center, New York, NY 10033, USA; Dino Ferrari Center, Department of Pathophysiology and Transplantation, University of Milan, Milan 20122, Italy; Pediatric Hepatology and Pediatric Liver Transplantation Unit, Bicêtre Hospital, Reference Center for Mitochondrial Disease, University of Paris-Saclay, Assistance Publique-Hôpitaux de Paris, Paris 94270, France; Center for Medical Genetics, Department of Metabolism, Chiba Children’s Hospital, Chiba 260-0842, Japan; Center for Medical Genetics, Department of Metabolism, Chiba Children’s Hospital, Chiba 260-0842, Japan; Diagnostics and Therapeutic of Intractable Diseases, Intractable Disease Research Center, Graduate School of Medicine, Juntendo University, Tokyo 113-8421, Japan; Diagnostics and Therapeutic of Intractable Diseases, Intractable Disease Research Center, Graduate School of Medicine, Juntendo University, Tokyo 113-8421, Japan; Department of Pediatrics, University Hospital Centre Zagreb and University of Zagreb, School of Medicine, Zagreb 10000, Croatia; Department of Pediatrics, University Hospital Centre Zagreb and University of Zagreb, School of Medicine, Zagreb 10000, Croatia; Clinic for Pediatrics, Division of Inherited Metabolic Disorders, Medical University of Innsbruck, 6020 Innsbruck, Austria; University Children’s Hospital, Paracelsus Medical University (PMU), 5020 Salzburg, Austria; Division of Metabolism, Bambino Gesù Children’s Hospital IRCCS, Rome 00165, Italy; Department of Biomedical and Neuromotor Sciences, University of Bologna, Bologna 40123, Italy; IRCCS Istituto di Scienze Neurologiche di Bologna, Programma di Neurogenetica, Bologna 40124, Italy; Dipartimento di Neuroscienze, Organi di Senso e Torace -Fondazione Policlinico Universitario Agostino Gemelli IRCCS, Rome 00136, Italy; Dipartimento Di Neuroscienze, Università Cattolica del Sacro Cuore, Rome 00168, Italy; Department of Pediatrics, University Medical Center Eppendorf, Hamburg 20246, Germany; Dipartimento di Neuroscienze, Organi di Senso e Torace -Fondazione Policlinico Universitario Agostino Gemelli IRCCS, Rome 00136, Italy; Dipartimento Di Neuroscienze, Università Cattolica del Sacro Cuore, Rome 00168, Italy; University Angers, Angers Hospital, INSERM, CNRS, MITOVASC, SFR ICAT, Angers F-49000, France; Centre de référence des maladies héréditaires du métabolisme, CHU la Timone Enfants, Marseille 13005, France; Fondazione IRCCS Ca’ Granda Ospedale Maggiore Policlinico, Regional Clinical Center for Expanded Newborn Screening, Milan 20122, Italy; Department of Pediatrics, Fondazione IRCCS San Gerardo dei Tintori, 20900 Monza, Italy; Unité de Gastroentérologie, Hépatologie, Nutrition et Maladies Héréditaires du Métabolisme, Hôpital des Enfants, CHU de Toulouse, Toulouse 31300, France; Division of Medical Genetics and Neurogenetics, Fondazione IRCCS Neurological Institute ‘C. Besta’, Milan 20133, Italy; Division of Neuropaediatrics and Paediatric Metabolic Medicine, Center for Paediatric and Adolescent Medicine, University Hospital Heidelberg, Heidelberg 69120, Germany; Department of Clinical and Experimental Medicine, Neurological Institute, University of Pisa & AOUP, Pisa 56126, Italy; Department of Clinical and Experimental Medicine, Neurological Institute, University of Pisa & AOUP, Pisa 56126, Italy; Fondazione IRCCS Ca’ Granda Ospedale Maggiore Policlinico, Regional Clinical Center for Expanded Newborn Screening, Milan 20122, Italy; Unit of Neurology and Neuromuscular Disorders, Department of Clinical and Experimental Medicine, University of Messina, Messina 98125, Italy; Department of Paediatrics, Medical Sciences Division, Oxford University, Oxford OX3 9DU, UK; Metabolic Unit, Meyer Children’s Hospital IRCCS, Florence 50139, Italy; Centre de référence des Maladies Mitochondriales, Service de Génétique Médicale, CHU de Nice, Université Côte d’Azur, CNRS, INSERM, IRCAN, Nice 06000, France; Division of Neuropaediatrics and Paediatric Metabolic Medicine, Center for Paediatric and Adolescent Medicine, University Hospital Heidelberg, Heidelberg 69120, Germany; Department of Clinical and Movement Neurosciences, UCL Queen Square Institute of Neurology, University College London, London WC1N 3BG, UK; Metabolic Clinic, Ruth Rappaport Children's Hospital, Rambam Health Care Campus, Haifa 3109601, Israel; The Ruth and Bruce Rappaport Faculty of Medicine, Technion-Israel Institute of Technology, Haifa 3109601, Israel; Metabolic Unit, Meyer Children’s Hospital IRCCS, Florence 50139, Italy; Department of Medical and Surgical Sciences, Alma Mater Studiorum University of Bologna, Bologna 40138, Italy; IRCCS Istituto delle Scienze Neurologiche, UO Neuropsichiatria dell’età Pediatrica di Bologna, Bologna 40124, Italy; Department of Biomedical and Neuromotor Sciences, University of Bologna, Bologna 40123, Italy; IRCCS Istituto di Scienze Neurologiche di Bologna, Programma di Neurogenetica, Bologna 40124, Italy; University Angers, Angers Hospital, INSERM, CNRS, MITOVASC, SFR ICAT, Angers F-49000, France; School of Medicine, Institute of Human Genetics, Technical University of Munich, Munich, 80333 Germany; Institute of Neurogenomics, Computational Health Center, Helmholtz Zentrum München, Neuherberg 80333, Germany; Department of Medical and Surgical Sciences, Alma Mater Studiorum University of Bologna, Bologna 40138, Italy; IRCCS Istituto delle Scienze Neurologiche, UO Neuropsichiatria dell’età Pediatrica di Bologna, Bologna 40124, Italy

**Keywords:** *DGUOK*, deoxyguanosine kinase, mitochondrial DNA, nucleosides, liver transplant

## Abstract

Autosomal recessive pathogenetic variants in the *DGUOK* gene cause deficiency of deoxyguanosine kinase activity and mitochondrial deoxynucleotides pool imbalance, consequently, leading to quantitative and/or qualitative impairment of mitochondrial DNA synthesis. Typically, patients present early-onset liver failure with or without neurological involvement and a clinical course rapidly progressing to death.

This is an international multicentre study aiming to provide a retrospective natural history of deoxyguanosine kinase deficient patients. A systematic literature review from January 2001 to June 2023 was conducted. Physicians of research centres or clinicians all around the world caring for previously reported patients were contacted to provide followup information or additional clinical, biochemical, histological/histochemical, and molecular genetics data for unreported cases with a confirmed molecular diagnosis of deoxyguanosine kinase deficiency.

A cohort of 202 genetically confirmed patients, 36 unreported, and 166 from a systematic literature review, were analyzed. Patients had a neonatal onset (≤ 1 month) in 55.7% of cases, infantile (>1 month and ≤ 1 year) in 32.3%, pediatric (>1 year and ≤18 years) in 2.5% and adult (>18 years) in 9.5%. Kaplan-Meier analysis showed statistically different survival rates (*P* < 0.0001) among the four age groups with the highest mortality for neonatal onset. Based on the clinical phenotype, we defined four different clinical subtypes: hepatocerebral (58.8%), isolated hepatopathy (21.9%), hepatomyoencephalopathy (9.6%), and isolated myopathy (9.6%). Muscle involvement was predominant in adult-onset cases whereas liver dysfunction causes morbidity and mortality in early-onset patients with a median survival of less than 1 year. No genotype–phenotype correlation was identified. Liver transplant significantly modified the survival rate in 26 treated patients when compared with untreated. Only six patients had additional mild neurological signs after liver transplant.

In conclusion, deoxyguanosine kinase deficiency is a disease spectrum with a prevalent liver and brain tissue specificity in neonatal and infantile-onset patients and muscle tissue specificity in adult-onset cases. Our study provides clinical, molecular genetics and biochemical data for early diagnosis, clinical trial planning and immediate intervention with liver transplant and/or nucleoside supplementation.

## Teaser

Manzoni *et al.* demonstrated Deoxyguanosine kinase deficiency as a tissue-specific mitochondrial DNA maintenance defect manifesting with rapidly progressive liver disease in early-onset patients and mild myopathy in adults. Early recognition is essential for immediate intervention with a Liver transplant, showing evidence of efficacy in the analysed cohort, and/or nucleoside supplementation.

## Graphical Abstract

**Graphical abstract:**
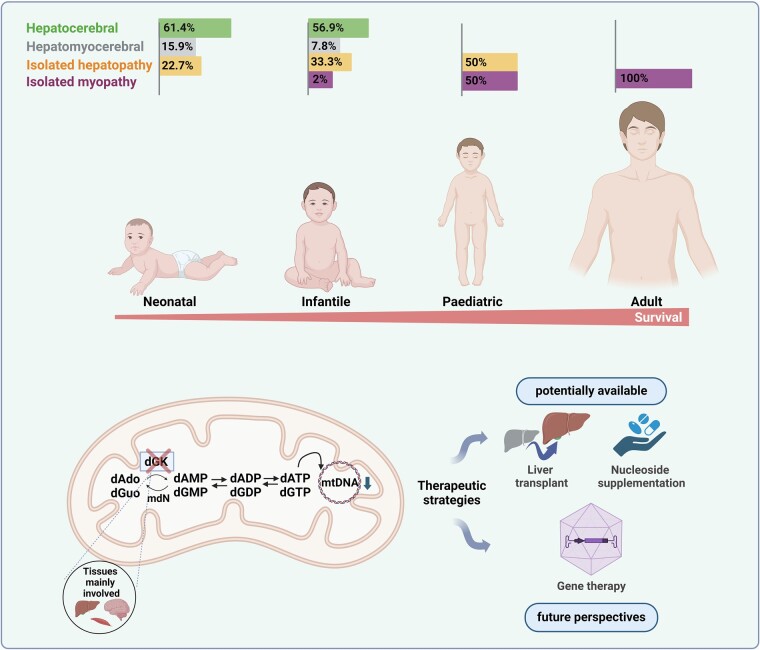

## Introduction

Mitochondrial DNA (mtDNA) is a circular double-stranded molecule of 16.6 kb present in multiple copies in cells and encoding for 13 protein subunits of the respiratory chain complexes, 2 rRNAs and 22 tRNAs.^1^ The maintenance of mtDNA requires a set of nuclear DNA (nDNA) encoded proteins, distinct from the nuclear replication machinery, responsible for the synthesis of the novel molecule, the supply of the deoxynucleotides (dNTP) pool through *de novo* synthesis or salvage pathway, the regulation and the packaging into the nucleoids.^2^ Defects in mtDNA maintenance are defined as defects of inter-genomics communication since pathogenic variants in nDNA genes affect quantitatively (depletion) and/or qualitatively (multiple deletions and points mutations) the integrity and copy number of mtDNA. Biochemically, they lead to reduced biosynthesis of mtDNA-encoded respiratory chain subunits and, consequently, to multiple oxidative phosphorylation (OXPHOS) activity defects. Clinically, they manifest as a spectrum of disorders going from tissue-specific diseases mainly affecting the liver, brain and muscle, to complex multi-organs disorders.^2^

Deoxyguanosine kinase (dGk) deficiency is one of the most common causes of hepatocerebral mtDNA depletion syndrome. Encoded by the nuclear gene *DGUOK*, Deoxyguanosine kinase (dGk) (OMIM #601465) is an enzyme of the mitochondrial dNTP pool salvage pathways that catalyzes the phosphorylation of deoxyguanosine (dG) and deoxyadenosine (dA) into their monophosphates. Those nucleotides are then converted into triphosphates (dGTP and dATP) and incorporated into the novel synthesized mtDNA molecule.^3^ Autosomal recessive pathogenetic variants in the *DGUOK* gene cause a lack of dGk enzyme activity and consequently unbalanced dNTP pool for the reduction of deoxy purines versus deoxy pyrimidines, ultimately leading to mtDNA depletion and/or multiple deletions.^4^ dGk deficiency was first described in 2001 by Mandel *et al.*,^5^ in 19 patients from three Druze kindreds, presenting an early onset (from birth to six months of age) of liver failure, severe failure to thrive, rotatory nystagmus, lactic acidosis, hypoglycemia, and neurological abnormalities. The disease was rapidly progressing to premature death before one year of age.^5^ Further case reports and case series studies contribute to identifying additional clinical presentations with early-onset isolated hepatopathy and mtDNA depletion^6,7^ or myopathy and mtDNA multiple deletions.^8^ There is no standard treatment for this devastating disorder. Liver transplant (LTx) has been controversial because of the complications related to the procedure, the risk of disease progression into multiorgan disease, and uncertainty about the development of a severe neurological phenotype despite liver transplantation. A comprehensive analysis of LTx outcomes is needed for identifying prognostic factors and giving recommendations based on evidence. Supplementation with nucleosides has been demonstrated safe and effective *in vivo* and *in vitro* models for nucleotides pool imbalance disorders^3,9,10^ giving the promise for translation to humans in clinical trials. In this view, natural history data are essential for identifying clinical endpoints, biomarkers, and parameters for trial design and readiness.

Our study aims to retrospectively analyze the largest cohort of dGk deficient patients including unpublished cases, for characterizing the clinical syndromes of dGk deficiency, identifying biochemical, molecular genetics, and histological/histochemical parameters for early diagnosis and prognosis, and understanding LTx outcome.

## Material and methods

This is a multicentre retrospective study focusing on patients affected by dGk deficiency. The study was conducted in accordance with both the Declarations of Helsinki and Istanbul. A systematic literature review was conducted using the terms ‘*DGUOK*’, ‘Deoxyguanosine kinase’, ‘dGk’ ‘Deoxyguanosine kinase deficiency’, ‘dGk deficiency’, ‘Mitochondrial DNA maintenance defects’ ‘Mitochondrial DNA depletion’ and ‘Mitochondrial deoxynucleotide pools imbalance’ from January 2001 to June 2023^4,5,7,8,11-59^ on PubMed. Manuscripts with available full text in English were considered. Physicians of research centres all around the world and clinicians caring for already reported patients were contacted to provide follow-up information or additional unreported cases with a confirmed molecular diagnosis of dGk deficiency. Informed consent for the anonymous publication of the patients’ clinical, biochemical, histological/histochemical and molecular genetics data was obtained from all study participants under the local ethics committee approval of the referring clinical centre at the time of the diagnostic workup. Collaborators from research centres in Austria (two), Croatia (two), France (four), Germany (three), Israel (one), Italy (nine), Japan (one), the UK (two) and the USA (one) were study contributors. Data collected include: demographics, survival, cause of death, age at onset and at the last follow-up, symptoms/signs of liver (jaundice, cholestasis, hepatomegaly, ascites), central and peripheral nervous system (central hypotonia, psychomotor delay, nystagmus, lethargy and peripheral neuropathy) or neuromuscular involvement (weakness/fatigue, ptosis, ophthalmoplegia, myalgia, dysphonia and dysphagia), systemic features (feeding difficulties, hypothermia and others), complications during the clinical course and need of medical/surgical procedures (ventilatory support, nutrition through nasogastric tube/gastrostomy and LTx), laboratory tests (transaminases levels, plasma ammonium and lactic acids, creatine phosphokinase (CK)), glycemia, plasma amino acids), molecular genetics results (*DGUOK* gene variants, mtDNA copy number and multiple deletions), electromyography (EMG) and nerve conduction (NCV) studies, brain magnetic resonance imaging (MRI) and brain spectroscopy (MRS), liver ultrasound and histological, biochemical and histochemical data from muscle and liver biological samples.

Molecular genetic studies of the DGUOK (NM_080916.3) gene were performed in reference centres by direct Sanger sequencing or next-generation sequencing of whole-exome or targeted genes panel library as previously described.^5,60,61^ Long PCR or Southern blot analysis was performed in DNA extracted from muscle homogenate to detect multiple mtDNA deletions and quantitative PCR for mtDNA copy number in available tissues (liver, muscle).^62,63^ dGk activity was measured in muscle or liver homogenate.^39^

All statistical analyses were performed using GraphPad Prism 8.0. Results were expressed as a ratio and percentage of available data. Clinical subgroup comparison and survival analysis were obtained by using the log-rank Mantel-Cox test.

## Results

### Clinical features

A cohort of 202 patients having a genetically confirmed diagnosis of dGk deficiency (88 male, 96 females, 18 not disclosed information) was studied (Supplementary Table 1). Data for 36 patients were not previously reported in the literature and were provided by study collaborators. Age at disease onset was available for 158 patients (44 N/A) ranging from birth to 69 years of age (median age: 7.2 months). Patients were stratified into four groups based on age at onset: 88/158 (55.7%) had a neonatal onset (≤1 month); 51/158 (32.3%) had an infantile-onset (>1 month and ≤1 year); 4/158 (2.5%) had a pediatric onset (>1 year and ≤18 years); and 15/158 (9.5%) had an adult-onset (>18 years).

**Figure 1:**
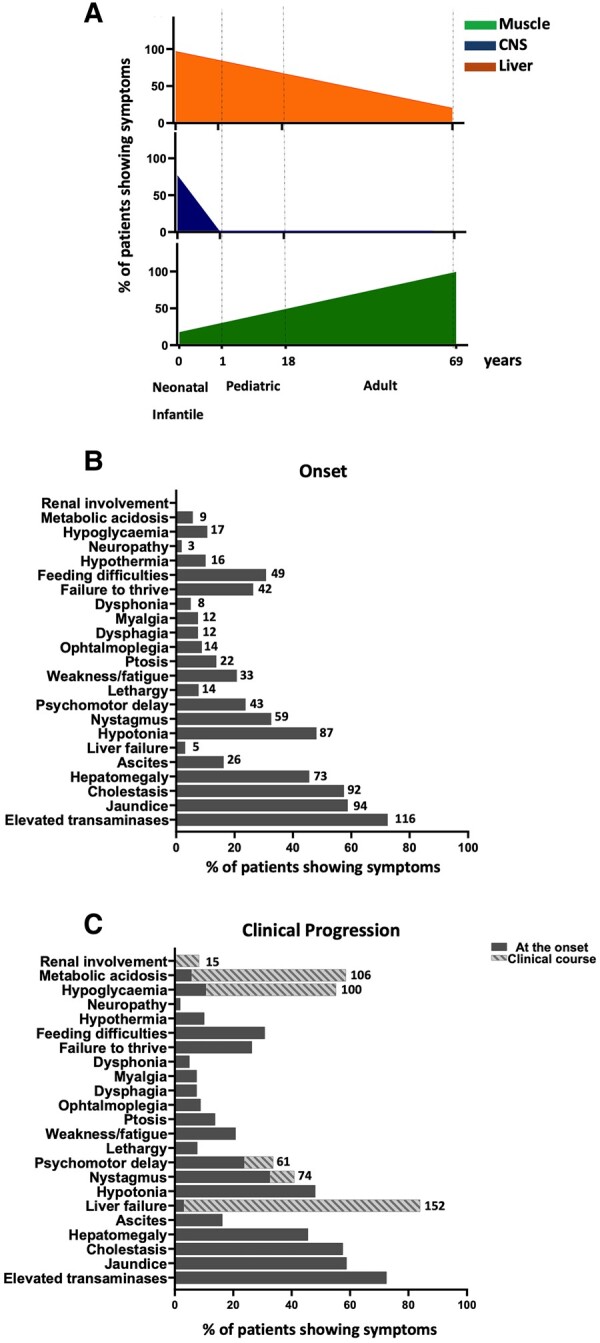
**Clinical manifestations of dGk deficient patients.** (**A**) Percentage of patients with muscular, central nervous system, or liver signs/symptoms at age of onset. Data were available for: 85/88 neonatal patients, 49/51 infantile patients, 4/4 pediatric patients and 15/15 adult patients. (**B, C**) Prevalence of symptoms in different organs or tissues at the disease onset (**B**) and during the clinical progression (**C**) expressed as a percentage. Data availability: 160/202 patients for liver symptoms, 181/202 patients for CNS symptoms, 159/202 patients for muscular symptoms. Follow-up data on the clinical course were available in 181/202. CNS: central nervous system.

The main organs/tissues affected by dGk deficiency were liver, central nervous system and muscle showing a different prevalence based on the age at onset. Liver involvement was more frequent in neonatal and infantile-onset while a tissue-specific muscle disease is associated with adult onset. CNS involvement was exclusively present in the early onset patients (Fig. 1A; Supplementary Table 2).

Signs and symptoms at onset and during the clinical course are graphically summarized in Fig. 1B and C. In detail, 141/160 (88.1%) presented liver involvement at onset and the most prominent sign was an increased level of alanine and aspartate transaminases (ALT/AST) described in 116/160 (72.5%) (ALT increase from 1.1-fold to 55.3; AST from 1.1 to 200.2). Patients also experienced cholestasis (92/160, 57.5%), jaundice (94/160, 58.8%), hepatomegaly (73/160, 45.6%), ascites (26/160, 16.3%) and liver failure (5/160, 3.1%). Data on liver signs/symptoms were not available for 42 patients. Liver ultrasound showed abnormalities in 40/50 patients: hepatomegaly in 15/40 (37.5%), hyperechogenicity in 10/40 (25%), ascites in 7/40 (17.5%), liver steatosis in 4/40 (10%), solid tumour in 4/40 (10%), patent abdominal vasculature in 2/40 (5%), other abnormalities not specified (globular or nodular morphology) in 5/40 (12.5%). In 10/50 cases liver ultrasound was normal.

Early central nervous system (CNS) symptoms were manifested in 123/181 (68%) presenting central hypotonia in 87/181 (48.1%), nystagmus in 59/181 (32.6%), psychomotor delay in 43/181 (23.8%) and lethargy in 14/181 (7.7%). In 58/181 cases there was no CNS involvement at disease onset. Data were not available in 21 patients. Brain MRI showed abnormal patterns in 25 out of 65 patients (38.5%). Specifically, cortical/subcortical atrophy was reported in 8/25 (32%), cerebellar atrophy in 3/25 (12%), white matter abnormalities in 9/25 (36%), focal lesions in 4/25 (16%), vasogenic pattern and cerebral edema in 3/25 (12%), grey matter abnormalities in 2/25 (8%). Brain MRS analysis showed a lactate peak in 6/8 patients.

Neuromuscular function was affected in 38/159 (23.9%) patients at disease onset. Data were not available in 43 cases. Weakness or fatigue was reported in 33/159 patients (20.8%), ptosis in 22/159 (13.8%), progressive external ophthalmoplegia in 14/159 (8.8%), myalgia in 12/159 (7.5%), dysphagia in 12/159 (7.5%) and dysphonia in 8/159 (5.0%). Electromyography (EMG) and nerve conduction velocity test (NCV) were normal in 6/19 (31.6%) while 10/19 (52.6%) showed a myogenic pattern and 3/19 (15.8%) a neurogenic pattern (only one with peripheral neuropathy).

Data on additional signs/symptoms at disease onset were available in 159/202 (47 N/A) and they were feeding difficulties (49/159, 30.8%), hypothermia (16/159, 10.1%), hypoglycemia (17/159, 10.7%) failure to thrive (42/159, 26.4%), metabolic acidosis (9/159, 5.7%), cardiomyopathy (1/159, 0,6%), cardiomegaly (1/159, 0,6%), retinal blindness (1/159, 0,6%), lactic acidosis (8/159, 5%) and neuropathy (3/159, 1.9%).

Follow-up data on the clinical course were available in 181/202 (21 N/A) showing disease progression in most cases. Metabolic acidosis complicated the clinical course in 106/181 (58.6%). The liver disease progressed into liver failure in 152/181 (84%). An increased number of patients presented psychomotor delay (61/181, 33.7%) and nystagmus (74/181, 40.9%). Renal involvement complicated the clinical course in 15/181 (8.3%). Ventilatory support was necessary for 9/181 (5%) patients and nutritional support was needed in 24/181 (13.2%) patients with a nasogastric tube in nineteen and gastrostomy in five.

Metabolic laboratory tests confirmed metabolic acidosis in 106/202 (52.5%) and hypoglycemia in 100/202 (49.5%) cases. Plasma amino acids showed abnormalities in 75 patients (37.1%, 127 N/A). Specifically, plasma tyrosine level was increased in 48/75 (64%), alanine in 21/75 (28%), methionine in 14/75 (18.7%), phenylalanine in 4/75 (5.3%) and glutamine in 3/75 (4%).

CK was increased from 1.2 to 11.8 fold in 25/48 (154 N/A). In addition, in two cases, one with the isolated hepatopathy and one with the hepatomyocerebral form, levels of 13 000 IU/L were reported during rhabdomyolysis episodes. Excluding those two patients, CK was increased from 1.4 to 6 fold in the hepatomyocerebral form, from 1.2 to 11.8 fold in the isolated myopathy, from 2.4 to 2.7 fold in the hepatocerebral, from 2.3 to 9.2 fold in isolated hepatopathy.

### Clinical classification

A total of 187 patients (15 N/A) were classified based on their clinical symptoms at-onset and during the disease course into the following four clinical subgroups: (i) hepatocerebral: patients showed signs/symptoms of the liver (jaundice, cholestasis, hepatomegaly, ascites, elevated transaminases, liver failure) and brain (central hypotonia, psychomotor delay, nystagmus, lethargy) involvement; (ii) hepatomyocerebral: in case of liver, brain and muscle involvement (weakness/fatigue, ptosis, ophthalmoplegia, myalgia, dysphonia, dysphagia); (iii) hepatic: isolated involvement of the liver; (iv) myopathic: isolated involvement of the muscle.

In our cohort of patients, 110/187 (58.8%) had a hepatocerebral form: 77/110 (70%) have had the complete form since the disease onset; 27/110 (24.5%) presented with neurological symptoms at onset and they further show signs of liver involvement; 6/110 (5.5%) presented with liver involvement and they further manifested neurological symptoms. The age of onset was only available for 83 patients in this subgroup (27 N/A). All of them had an early onset: neonatal in 54/83 (65.1%) while infantile in 29/83 (34.9%). Additional symptoms were: metabolic acidosis in 75/110 (68.2%), hypoglycemia in 67/110 (60.9%), failure to thrive in 40/110 (36.4%), feeding difficulties in 33/110 (30%), renal involvement in 10/110 (9.1%), hypothermia in 10/110 (9.1%), neuropathy in 2/110 (1.8%), cardiomyopathy in 1/110 (0.9%), cardiomegaly in 1/110 (0.9%), retinal blindness in 1/110 (0.9%). Data on mortality revealed that 99/106 (93.4%) (4 N/A) patients died at an average age of 6.79 months (ranging from less than one month to 1.5 years), while 7/106 (6.6%) were still alive at the last follow-up, with an age that ranged from less than a month to seventeen years. Data on mortality were not available in four patients.

Isolated liver involvement (hepatic form) was reported in 41/187 (21.9%) cases. Age of onset was ranging from neonatal (20/39, 51.3%) to infantile (17/39, 43.6%) to pediatric-onset (2/39, 5.1%). No adult onset was recorded (2 N/A). Additional symptoms were: hypoglycemia in 19/40 (47.5%), metabolic acidosis in 14/40 (35%), feeding difficulties in 3/40 (7.5%), renal involvement in 3/40 (7.5%), hypothermia in 3/40 (7.5%), failure to thrive in 1/40 (2.5%) (1 N/A). Data on mortality were available for 40 individuals (1 N/A): 25/40 (62.5%) died at an average age of 6.2 months (ranged from less than 1 month to 2.58 years), while 15/40 (37.5%) survived at an average age of 6 years (range from 1 month to 19 years).

Hepatomyocerebral form was present in 18/187 (9.6%) patients: 14/18 (77.8%) had a neonatal onset and 4/18 (22.2%) had an infantile onset. Additional symptoms were: metabolic acidosis in 16/18 (88.9%), hypoglycemia in 14/18 (77.8%), feeding difficulties in 11/18 (61.1%), hypothermia in 3/18 (16.7%), renal involvement in 2/18 (11.1%), failure to thrive in 1/18 (5.6%). The clinical course was rapidly progressing to premature death in 13 out of 18 (72.2%) at an average age of 6.8 months whereas 5/18 (27.8%) patients were still alive at the last follow-up (age range from 4 months to 26 years).

The myopathic form was present in 18/187 (9.6%) cases: 15/18 (83.3%) had an adult-onset, 2/18 had a pediatric onset (11.1%) and 1/18 (5.6%) presented an infantile-onset. Additional symptoms were: feeding difficulties in 2/18 (11.1%), metabolic acidosis in 1/18 (5.6%), neuropathy in 1/18 (5.6%), and parkinsonism in 1/18 (5.6%). At the last follow-up, 12/14 (85.7%) subjects were still alive (average age 51.1 years). Two patients (14.3%) died respectively at the age of 65 and 69 years (4 N/A).

Additional data are summarized in Supplementary Table 3.

### Survival rate

At the last follow-up, 139 patients were deceased (data not available for 24 patients).

**Figure 2:**
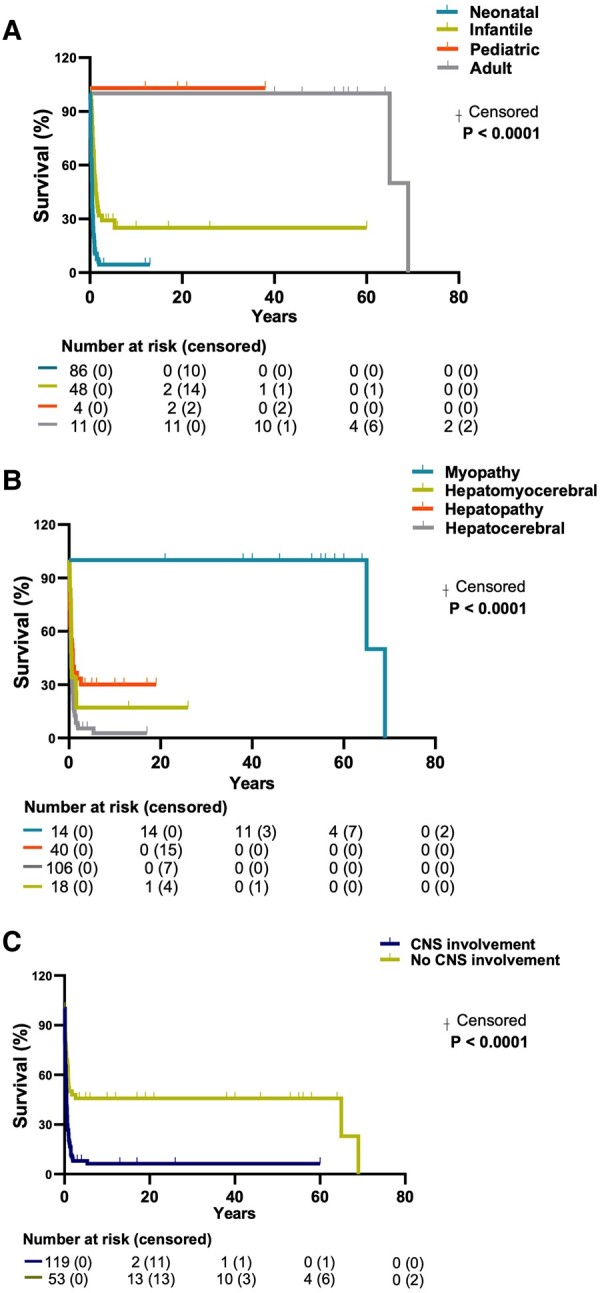
**Kaplan-Meier analysis of dGk deficiency mortality.** (**A**) Survival curve stratified for age at onset (*n* = 149) and (**B**) clinical forms (*n* = 178); (**C**) Survival curve in dGk deficient patients with or without CNS involvement (*n* = 172). Survival analysis was assessed by log-rank Mantel-Cox test. Numbers at risk are specified below each panel. When statistically significant, *P* values are indicated in the figure. CNS: central nervous system.

Kaplan-Meier analysis showed a statistically significant difference in the survival rate among the age-at-onset subgroups (158/202 censored data, 44 N/A) (*P* < 0.0001 by Mantel-Cox test) with the highest mortality for the neonatal group (Fig. 2A). Median survival was 0.5 years for the neonatal onset, 1.17 years for the infantile-onset and 67 years for the adult-onset group. Median survival for the pediatric-onset was not directly estimable because of the low number of censored patients.

Kaplan-Meier analysis comparing the survival rate of the different clinical forms (187/202) (Fig. 2B) showed a statistically significant difference between the myopathic form and all other clinical phenotypes (*P* < 0.0001 by Mantel-Cox test). The median survival was less than 1.0 years for the hepatomyocerebral (0.50), the hepatocerebral (0.42) and the hepatic form (0.75), while for the myopathic form was 67 years.

We analyzed the presence of neurological signs/symptoms as a negative predictor for survival in a total cohort of patients. Among 119 patients who had neurological signs/symptoms, 106 patients were deceased at the last follow-up (89.1%). The difference between patients with and without neurological signs was statistically significant (*P* < 0.0001, by Mantel-Cox test, Fig. 2C). The median survival was 0.5 years for patients with neurological involvement and 1.75 years for patients without neurological symptoms. However, this data include patients with isolated myopathy who had a normal life span compared to other patients’ subgroups. There was no difference in survival rate when comparing patients with normal brain MRI versus patients presenting abnormalities (Supplementary Fig. 1).

Data on the cause of death were available in 112 out of 139 deceased patients (27 N/A). The most frequent cause of death was liver failure, reported in 81/112 patients (72.3%), followed by multi-organ failure (MOF) in 12/112 (10.7%), encephalopathy in 5/112 (4.5%), cardiac arrest in 4/112 (3.6%), gastrointestinal bleeding in 5/112 (4.5%), pulmonary arterial hypertension in 4/112 (3.6%), LTx complications in 2/112 (1.8%), shock in 2/112 (1.8%), peritonitis in 1/112 (0.9%), hypertensive crisis in 1/112 (0.9%), meningococcus meningitis in 1/112 (0.9%), Non-Hodgkin lymphoma in 1/112 (0.9%) and metabolic acidosis in 1/112 (0.9%).

### LTx outcome

**Figure 3:**
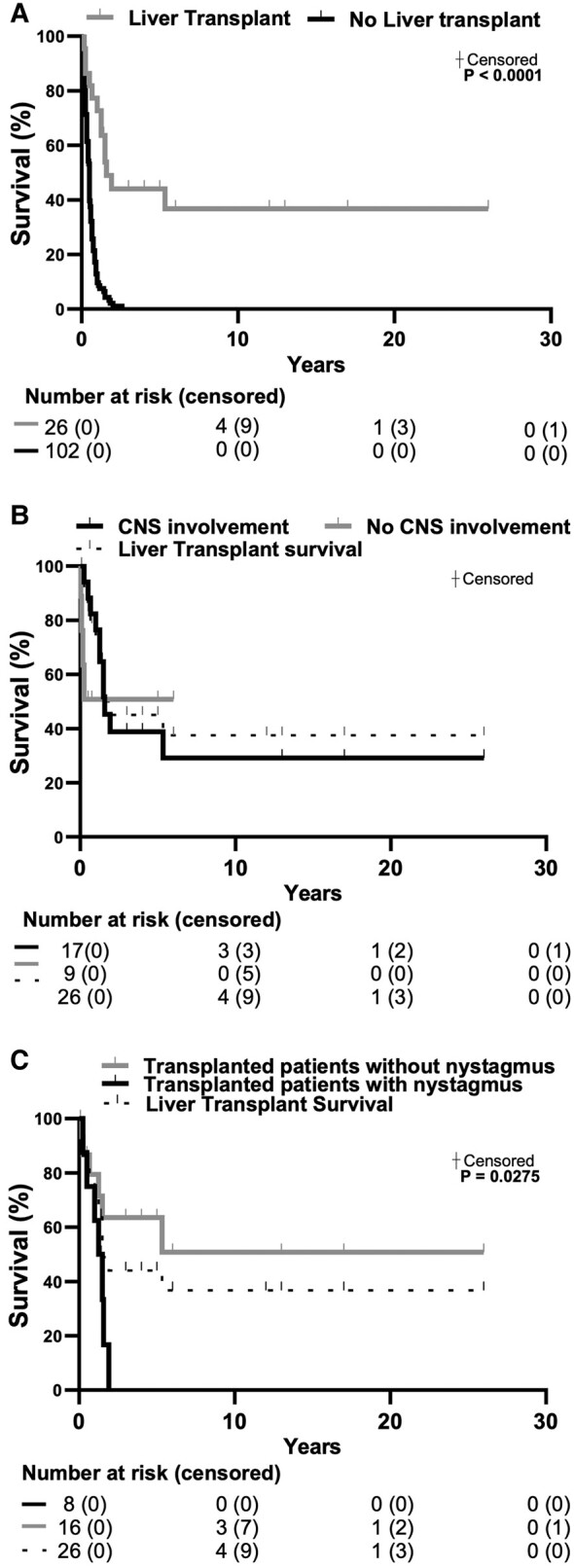
**LTx outcome:** (**A**) Survival curves comparing LTx versus untreated patients (*n* = 128). (**B**) Survival curve comparing subgroups of LTx patients presenting or not CNS involvement (*n* = 26) and (**C**) specifically with or without nystagmus (*n* = 24). Survival analysis was assessed by log-rank Mantel-Cox test. Numbers at risk are specified below each panel. When statistically significant, *P* values are indicated in the figure. CNS, central nervous system; LTx, liver transplant.

LTx was performed in 26 patients: 14 with the hepatocerebral form, 9 with isolated hepatopathy, and 3 with hepatomyocerebral form. Kaplan-Meier analysis demonstrated a statistically significant difference in survival time when comparing treated versus untreated patients (*P* < 0.0001) (Fig. 3A). The median survival was 1.92 years in the overall cohort of transplanted patients (ranging from 1 month to 26 years) versus 0.5 years in untreated patients (ranging from 1.4 months to 6 months). Five patients died soon after the procedure. At one year of follow-up post-transplant data were available in 8 patients that were alive and among them four patients had a long-term survival of 10 years; 5 patients were reported alive after the transplant but the length of survival is not available. Neurological symptoms were recorded in 17/26 (65.4%) transplanted patients. We compared the survival rate of individuals with or without neurological involvement but there was no statistically significant difference (Kaplan-Meier, *P* = 0.5410) (Fig. 3B). We enquired if the presence of MRI abnormalities or specific signs/symptoms such as nystagmus, central hypotonia or psychomotor delay was a negative predictor for survival after LTx and Kaplan-Meier analysis showed no difference in patients with MRI abnormalities, psychomotor delay, or central hypotonia at disease onset in survival after LTx (Supplementary Fig. 2A–C). However, patients with nystagmus at disease onset had a negative outcome after LTx with a statistically significant difference in survival rate (Kaplan-Meier, *P* = 0.0275) (Fig. 3C). We reviewed the neurological outcome among survivors: six patients developed additional neurological signs after LTx which were psychomotor delay (4), nystagmus (3), and hyposthenia (1). An improvement in neurological involvement was recorded in one. Causes of death in transplanted patients were pulmonary hypertension in 4/13 (30.8%), LTx complications in 2/13 (15.4%), MOF in 1/13 patients (7.7%), hypertensive crisis in 1/13 patients (7.7%), peritonitis in 1/13 patients (7.7%), cardiac arrest in 1/13 patients (7.7%), cardiac tamponade in 1/13 patients (7.7%), liver failure in 1/13 patients (7.7%), encephalopathy in 1/13 patients (7.7%).

### Molecular genetics and biochemical studies

mtDNA copy number was measured in DNA extracted from liver biopsy samples in 58/202 patients (141 N/A) (median value: 15.8% ranging from 0% to 98%) presenting early onset of hepatocerebral form in 31/58 (53.4%), hepatopathy in 16/58 (27.6%), hepatomyocerebral in 10/56 (17.2%) (1 N/A). mtDNA depletion was found in all of them.

mtDNA copy number was measured in DNA extracted from muscle biopsy samples in 30/202. Depletion was found in 20/30 (66.7%) (median value: 30.7% ranging from 6% to 89%) presenting an hepatocerebral form in 9/20 (45%), hepatomyocerebral in 6/20 (30%), hepatopathy in 4/20 (20%) and myopathy in 1/20 (5%). Normal mtDNA copy number was found instead in 4/10 patients with hepatocerebral form, 5/10 with myopathy and 1/10 with isolated hepatopathy. The presence of multiple mtDNA deletions was analyzed in 58 patients: 13 cases with myopathic phenotype and one with hepatopathy had multiple deletions. Only one case had both depletion and deletions with adult-onset myopathy.^11^

**Figure 4:**
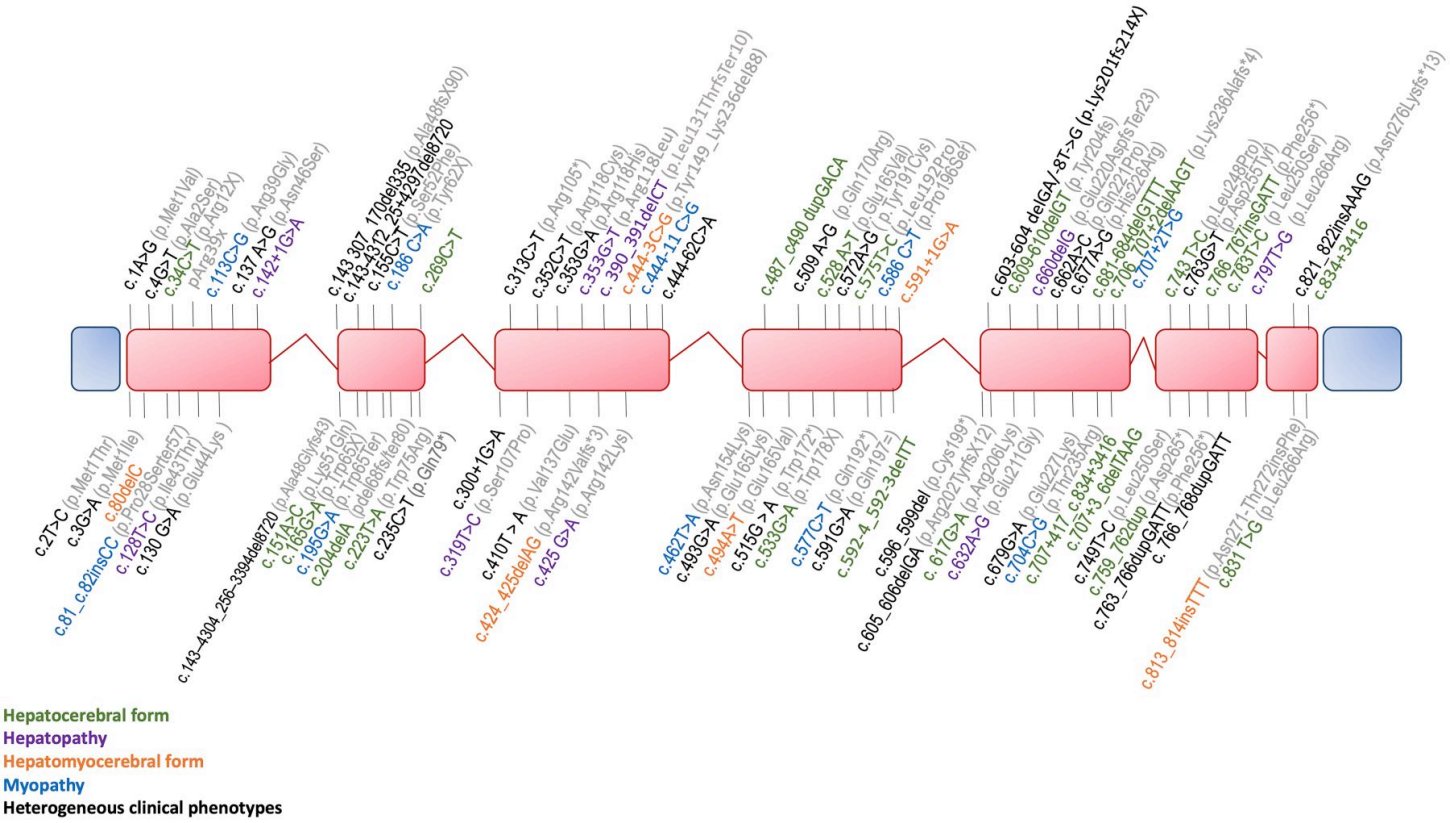
***DGUOK* disease-causing variants:** graphical representation of *DGUOK* gene showing identified pathogenic variants in the coding and splice-site regions (NM_080916.3). Exons are marked in red boxes. Protein changes are colour-coded based on the clinical form.

Genetic analyses allow the identification of 82 pathogenic variants (Fig. 4). There was no clear genotype–phenotype correlation. The most frequent variants were: a) c.255del (p.Ala86Profs*13), located in exon 2, and identified in 21/202 (10.4%) patients from five unrelated families, presenting the hepatocerebral form in 20 cases and the hepatomyocerebral form in one case; b) c.3G > A (p.Met1Ile), located in exon 1 and identified in 20/202 (9.9%) patients from 19 unrelated families; c) c.763_766dupGATT (p.Phe256*), located in exon 6 and identified in 18/202 (8.9%) patients from 12 unrelated families (3 N/A). There was no specific clinical syndrome associated with these latter variants.

### Muscle and liver biopsy

Data on histological analysis of liver and muscle biopsy samples were collected respectively for 87 and 45 patients. Liver specimen analyses revealed steatosis in 61/87 (70.1%) patients, cholestasis in 52/87 (59.8%), fibrosis in 49/87 (56.3%), cirrhosis in 29/87 (33.3%), siderosis in 23/87 (26.4%) and necrosis in 16/87 (18.4%). Muscle biopsy analyses demonstrated atrophic myofibres in 4/45 (8.9%) patients, sarcolemmal mitochondrial aggregates in 3/45 (6.7%), rare central nuclei and splitting in 2/45 (4.4%), fibrosis in 1/45 (2.2%). Histochemical analysis of muscle biopsy showed cytochrome c oxidase (COX) deficient muscular fibres in 19/45 (42.2%) patients, ragged red fibres in 13/45 (28.9%), lipid storage in 6/45 (13.3%) and succinate dehydrogenase (SDH) positive fibres in 7/45 (15.6%).

Biochemical analysis of respiratory chain complex activities in liver biopsy samples was available in 64 patients: 59/64 (92.2%) had multiple OXPHOS defects, one patient had isolated Complex I deficiency (1.6%), one patient had isolated Complex III deficiency (1.6%), two isolated Complex IV deficiency (3.1%) and two had normal activities (3.1%). Citrate synthase level was available in 15 patients and was increased in 13/15 (86.7%), normal in the remaining two patients.

Biochemical analysis of respiratory chain complex activities in muscle homogenate was available in 44/202 (21.8%). Multiple OXPHOS deficiency was present in 14/44 (31.8%) patients, isolated Complex I deficiency in 4/44 (9.1%), isolated Complex IV deficiency in 4/44 (9.1%), isolated Complex V deficiency in 1/44 (2.3%), normal activity in 21/44 (47.75%). Citrate synthase level was available in 11 patients and was increased in 7/11 (63.6%) and reduced in 2/11 (22.2%), while it was normal in two patients (22.2%).

Biochemical analysis of respiratory chain complex activities in patients’ fibroblast cell lines was available in 9/202 (4.46%) revealing multiple OXPHOS deficiency in 4/9 (44.4%) patients, isolated complex IV deficiency in 1/9 (11.1%) and normal activities in 4/9 (44.4%).

dGk activity was measured in muscle in 4 patients and liver in 2 patients showing a range of residual activity respectively from 19% to 45% and from 0.5% to 27%.

## Discussion

Mitochondrial disorders associated with mtDNA instability are due to autosomal recessive (*ABAT, AGK, ANT1, ATAD3A, DGUOK, DNA2, DNM1L, DTYMK, FBXL4, MFN2, MGME1, MPV17, MSTO1, OPA1, POLG1, POLG2, RNASEH1, RRM2B, SSBP1, SUCLA2, SUCLG1, TFAM, TK2, TYMP, TWNK*) or dominant (*ANT1, ATAD3A, DNA2, DNM1L, SSBP1, OPA1, POLG1, POLG2, RRM2B, TWNK*) variants in nDNA genes encoding proteins playing role in the maintenance pathway.^64-68^ The molecular genetics hallmark of all defects is a severe reduction of mtDNA copy number (depletion) in early-onset cases or the presence of multiple mtDNA deletions in adult-onset cases.^8^ The coexistence of mtDNA depletion, multiple deletions, and point mutations is an exclusive characteristic of mitochondrial neuro gastrointestinal encephalomyopathy (MNGIE)^69^ while a moderate reduction of mtDNA copy number together with mtDNA multiple deletions has been demonstrated in juvenile-onset thymidine kinase 2 deficiency (TK2) myopathy.^70^ Clinically, they manifest with a prevalent tissue-specificity in early onset cases to liver (*DGUOK, MPV17, POLG1, POLG2, TFAM*), brain (*ABAT, DNM1L, DTYMK, POLG1, SUCLA2, SUCLG1*), heart (*AGK, ANT1*) and muscle (*TK2*), although a multiorgan involvement is also described.^66,71^ Adult-onset syndrome is mainly characterized by progressive external ophthalmoplegia with/without proximal or respiratory muscle weakness (*ANT1, DGUOK, DNA2, MGME1, POLG1, POLG2, RNASEH1, RRM2B, TK2, TWNK*).^71^

Autosomal recessive variants in the *DGUOK* gene cause dGk deficiency and consequently an unbalanced supply of purine to mtDNA synthesis. Although dGk deficiency is a rare disease it represents a frequent metabolic cause of liver failure in infancy. A previous study by Al-Hussaini *et al.*^7^ in 2014 demonstrated that *DGUOK* defect was the cause of liver failure and cholestasis in 22% of a cohort of 450 infantile patients and it was associated with poor prognosis. Here we presented data on the largest cohort of 202 patients genetically confirmed dGk deficient, 36 of which were not previously reported in the literature.

Our study demonstrated that dGk deficiency is predominantly an early-onset liver and brain tissue-specific disorder. Liver disease was present in 88% of patients presenting jaundice, elevated transaminase, hepatomegaly or ascites before one year of age and 84% of patients progressed to liver failure within a few weeks or months. Neurological signs were also present in 68% of patients and characterized by a variable association of central hypotonia, psychomotor delay and nystagmus. However, it is not possible to exclude that those symptoms were secondary to the liver disease, general metabolic condition or chronic illness and associated hospitalizations of the patients. Brain MRI investigation was normal in more than 50% (40/65) and showed variable abnormalities in others as cortical/subcortical atrophy (8/25, 32%), cerebellar atrophy (3/25, 12%), white matter abnormalities (9/25, 36%), focal lesions (4/25, 16%), vasogenic pattern and cerebral edema (3/25, 12%) and grey matter abnormalities (2/25, 8%). CNS involvement was not progressive during the clinical course in all patients except three who died of encephalopathy.

Pediatric-onset was sporadic with only four patients (2.5%) presenting symptoms between one and eighteen years of age. An adult-onset disease instead was reported in 9.5% of patients with a muscle tissue-specificity mainly characterized by proximal muscle weakness and fatigue with/without progressive external ophthalmoplegia (PEO). Interestingly, in one case a neurological involvement with parkinsonism was reported.^11^

Patients presented additional symptoms related to the general metabolic condition such as failure to thrive, hypoglycemia, and hypothermia. Additional organs/tissues comprised 15 patients with renal involvement, two cases with cardiomyopathy, and one case with retinal blindness. Metabolic acidosis was an acute event in the clinical course of 52.5% of patients. Feeding difficulties were reported in 30.8% of patients but required nutritional support in only 13.2% of patients with a nasogastric tube in 19 cases and gastrostomy in 5 cases. Respiratory insufficiency was not a major cause of morbidity in our cohort with only nine patients requiring ventilator support (5%).

Diagnosis of dGk deficiency is challenged by clinical, histological and biochemical features overlapping other metabolic and mitochondrial disorders. In our cohort of patients plasma amino acids revealed a high level of tyrosine in 64% of cases posing differential diagnosis with Tyrosinemia type I, a rare autosomal recessive disorder characterized by progressive liver disease and a secondary renal tubular dysfunction.^72^ Succinylacetone was reported in trace or absent in urine when measured.^12,13,26,27,36^ However, a diet restricted from tyrosine and phenylalanine was considered in some cases before the genetic diagnosis.^12,36^ Genetic tests of the *DGUOK* gene in patients presenting a high level of tyrosine at newborn screening should be considered for preventing disease progression with early intervention with LTx and/or nucleoside supplementation. Liver ultrasound and histological analyses revealed in 26.4% of patients the presence of iron deposit/overload leading to misdiagnosis of hemochromatosis.^12-15^ In a limited number of cases, there were also increased levels of plasma ferritin^4,12,15,28^ and one patient was treated with iron chelant.^13^ Hepatosplenomegaly and hypoglycemia were responsible for a misdiagnosis of glycogen storage disease in a 4-month-old Turkish patient.^27^ Other differential diagnoses that have been considered in the diagnostic workup were: homocystinuria, 2-CH3-3-hydroxybutyric aciduria, galactosemia, hereditary fructose intolerance, citrullinemia type II, biotinidase deficiency, Wilson disease and lysosomal storage disorders.^14^ Adult myopathy may instead overlap with oculopharyngeal myopathy, myasthenia gravis, and other genetically inherited progressive external ophthalmoplegia. Differential diagnosis should also be considered with other mitochondrial disorders causing liver failure (*MPV17, POLG1, POLG2, TFAM, TWNK, TRMU*). Genetics tests with either targeted gene panels or whole exome sequencing and, whenever possible, tissue-specific mtDNA quantity and integrity analysis should be performed to achieve the diagnosis of dGk deficiency.

Important features enable us to stratify patients regarding the disease morbidity and mortality: age at onset, main tissue/organ presenting signs and symptoms, and survival. Patients with neonatal and infantile-onset onset had the highest mortality with a median survival respectively of 0.5 and 1.2 years whereas adult-onset patients had a normal life span with a median survival of 67 years. We have confirmed the phenotypic heterogeneity of dGk deficiency and classified the disease into four major clinical forms (hepatocerebral, hepatomyocerebral, isolated hepatopathy, and isolated myopathy) presenting as a continuum spectrum from the neonatal to the adult onset. The most frequent clinical forms were hepatocebral in 58.8% of patients and isolated hepatopathy in 21.9%. Only a small subgroup of patients presented muscle symptoms: 9.6% with hepatomyocerebral form and 9.6% with isolated myopathy. This latter clinical form might be underdiagnosed since it has been described more recently and clinical symptoms are mild and overlapping with other mitochondrial neuromuscular disorders. We have observed a similar phenomenon in TK2 deficiency, in which the late-onset adult form had an increased prevalence when the disease classification was reported.^70,73^

Our data demonstrated that the hepatocerebral form had the highest mortality with 93.4% of patients dying at an average age of 6.85 months (ranging from less than one month to 5.3 years). Patients with isolated myopathy had a better outcome with 85.7% of patients still alive at the last follow-up (average age 51.1 years) and only two patients died at the age of 65 and 69 years. We enquired if the presence of brain MRI abnormalities was a negative prognostic factor, but we found no difference in the survival rate.

The most common cause of death was liver failure in 72.3% of patients followed by MOF in 10.7% of cases. Other causes of death were sporadic: encephalopathy (5), cardiac arrest (4), gastrointestinal bleeding (5), pulmonary arterial hypertension (4), LTx complications (2), shock (2), peritonitis (1), hypertensive crisis (1), meningococcus meningitis (1), Non-Hodgkin lymphoma (1), metabolic acidosis (1).

Molecular genetics studies demonstrated that mtDNA depletion was present in all liver samples analyzed in patients presenting signs/symptoms of hepatic disease whereas muscle samples analysis showed a reduction of mtDNA copy number in only 66% of the cases. The level of mtDNA copy number in muscle can vary from normal to severely decreased in patients with hepatopathy as already indicated by Al-Hussaini *et al.*^7^ in 2014. Additionally, it might change during the follow-up while the disease progresses to muscle involvement as reported since the first report by Mandel *et al.*^5^ in 2001. Our data together with the literature review suggest that the most affected tissue should be analyzed for the diagnosis. Multiple deletions of mtDNA were found instead in 14 out of 58 muscle samples of our cohort and associated with adult myopathy in all except for one patient with isolated hepatopathy. Reduction in mtDNA copy number around 50–60% and multiple deletions were reported in two patients.^11^ Data on the coexistence of moderate reduction in mtDNA copy number and multiple deletions might be underestimated since muscle samples were not analyzed for both defects in all late-onset patients. Taken together our results confirmed the tissue-specificity of dGk deficiency also at the molecular-genetics level. This highlights that muscle biopsy is not informative in patients with hepatopathy and it suggests that mtDNA copy number analysis is the gold standard for the diagnosis in liver biopsy in patients with hepatopathy as well as the mtDNA multiple deletions in muscle patients with isolated myopathy.

Mitochondrial respiratory chain activities confirmed the presence of multiple deficiencies in liver and muscle homogenate in most patients. Only sporadic cases showed isolated complex I, III, IV or V deficiency.

A total of 82 distinct pathogenic variants have been found in the DGUOK gene with hot-spot regions in exons 1, 2, and 6. The most frequent variants were c.255del (p.Ala86Profs*13), identified in 10.4% of patients presenting hepatocerebral form; c.3G > A (p.Met1Ile) identified in 9.4% of patients and c.763_766dupGATT (p.Phe256*) in 8.9% patients, both not associated with a specific clinical syndrome. We did not find any genotype–phenotype correlation, contrary to a previous study by Dimmock *et al.*^4^ in 2008. Similar to other diseases in the same pathways, there was inter- and intrafamilial variability for the type and severity of clinical symptoms.

Inborn errors of metabolism are the second most common indication for LTx in the pediatric population with an excellent survival outcome (>95% at one year, between 80% and 90% at 10 years).^74^ In mitochondrial hepatopathies, LTx has been considered controversial due to unpredictable outcomes and ethical problems in allocating resources to children that might present extrahepatic involvement and/or possible neurological deterioration. A first preliminary study in a cohort of dGk deficient patients by Dimmock *et al.*^75^ in 2008 suggests a long-term survival in the absence of profound central hypotonia, significant psychomotor retardation or nystagmus but further case reports or series studies argue the benefit of LTx even in the presence of mild neurological symptoms.^24^ Al-Hussaini *et al.*^7^ in 2014 reported four patients that had LTx and developed neurologically normal after 2–4.5 years after the transplant.^7^ Ronchi *et al.*^8^ in 2012 identified a patient who had LTx at an infantile age and was free from symptoms up to adult age when she presented mild myopathy with isolated episodes of rhabdomyolysis.^8^ In 2020, Jankowska *et al.*^24^ analyzed a cohort of 20 patients, two not previously reported and 18 from the literature review that had LTx at the age of 1–18 months and the survival rate was 50% with the cause of death related to procedure complications in all the patients. Neurological outcome was overall positive in five patients with good psychomotor development and/or mild central hypotonia 2.5–17 years after LTx, one patient with moderate cognitive impairment and recurrent episodic rhabdomyolysis, and only one patient with severe psychomotor retardation one year after LTx. The authors concluded that LTx was not contraindicated in patients without or with minimal neurologic abnormalities.^24^ We contributed to this analysis and in our cohort, 26 patients underwent LTx. Kaplan-Meier analysis of survival rate demonstrated a statistically significant difference among treated and untreated patients with a survival rate ranging from 1 month to 26 years in transplanted patients whereas it ranged from one to 6 months in non-transplanted patients. The number of patients that died because of the procedures was higher than other metabolic disorders but the outcome at one year in the survival population was consistently good. The presence of neurological involvement was not a negative predictor of survival rate. However, when we analysed psychomotor delay or central hypotonia or nystagmus alone, we found a statistically significant difference in survival in patients presenting nystagmus at disease onset. We can speculate that nystagmus is a sign of a more progressive disorder but further observation in a prospective study is needed to support this hypothesis including a more extensive investigation of its aetiology. Therefore, although our data suggest nystagmus as a negative predictor for the LTx outcome, caution must be posed in determining the decision on only a single sign. Encephalopathy was the cause of death in only one case out of 26 patients. Among survivors, six patients developed additional mild neurological signs which were psychomotor delay (4), nystagmus (3) and hyposthenia (1). An improvement in neurological involvement was recorded in one case.

Overall, our data suggest that dGk-deficient patients may benefit from LTx and the decision should be made on the risk assessments of the potential complications related to the procedures or the general conditions of the patients. LTx may save patients from premature death and make them eligible for further treatment approaches such as nucleoside supplementation therapies. *In vivo* and *in vitro* data on nucleoside supplementation have demonstrated phenotypically, molecular genetics, and biochemically efficacy and safety in mitochondrial dNTP pool imbalance disorders.^3,9^ Zebrafish dguok−/− model reproduces the human disease showing a lack of enzyme activity and mtDNA depletion, the latter rescued by purine supplementation in adult fish.^10^ Nucleoside treatment has been already translated in Phase 2 open-label clinical trials (NCT03845712, NCT03701568) for compassionate use in patients with TK2 deficiency with a dramatic impact on the natural history of the disease.^76^ Nucleosides can cross the blood-brain barrier and could have an impact on CNS involvement. Therefore, a combination of LTx and nucleoside supplementation may be a potentially effective therapy for dGk-deficient patients presenting with liver disease. Gene therapy with adeno-associated viral vector transduction or gene editing with CRISPR/Cas9 technology holds the promise to treat rare genetic diseases but no data are currently available in pre-clinical dGk deficient models.

In conclusion, dGk deficiency is a tissue-specific mtDNA maintenance defect manifesting with rapid and progressive liver disease in early-onset patients and mild myopathy in adult-onset patients. Our study provides diagnostic clinical and molecular genetic criteria of the disease clinical forms, identifies morbidity and mortality predictors and describes LTx outcomes. Early recognition may prompt immediate intervention before the disease progresses with LTx and/or nucleoside supplementation.

## Supplementary Material

fcae160_Supplementary_Data

## Data Availability

The anonymized data can be accessed on reasonable request addressed to the corresponding author.

## References

[1] DiMauro S . Mitochondrial encephalomyopathies–fifty years on: The Robert Wartenberg lecture. Neurology. 2013;81(3):281–291. 23858410 10.1212/WNL.0b013e31829bfe89 PMC3959764

[2] Spinazzola A, Zeviani M. Disorders from perturbations of nuclear-mitochondrial intergenomic cross-talk. J Intern Med. 2009;265(2):174–192. 19192035 10.1111/j.1365-2796.2008.02059.x

[3] Camara Y, Gonzalez-Vioque E, Scarpelli M, et al Administration of deoxyribonucleosides or inhibition of their catabolism as a pharmacological approach for mitochondrial DNA depletion syndrome. Hum Mol Genet. 2014;23(9):2459–2467. 24362886 10.1093/hmg/ddt641 PMC6281351

[4] Dimmock DP, Zhang Q, Dionisi-Vici C, et al Clinical and molecular features of mitochondrial DNA depletion due to mutations in deoxyguanosine kinase. Hum Mutat. 2008;29(2):330–331. 10.1002/humu.9519 18205204

[5] Mandel H, Szargel R, Labay V, et al The deoxyguanosine kinase gene is mutated in individuals with depleted hepatocerebral mitochondrial DNA. Nat Genet. 2001;29(3):337–341. 11687800 10.1038/ng746

[6] El-Hattab AW, Scaglia F, Wong LJ. Deoxyguanosine kinase deficiency. In: Adam MP, Ardinger HH, Pagon RA, et al, eds. GeneReviews® [Internet]. Seattle (WA): University of Washington; 1993–2024. 20301766

[7] Al-Hussaini A, Faqeih E, El-Hattab AW, et al Clinical and molecular characteristics of mitochondrial DNA depletion syndrome associated with neonatal cholestasis and liver failure. J Pediatr. 2014;164(3):553–9.e2. 24321534 10.1016/j.jpeds.2013.10.082

[8] Ronchi D, Garone C, Bordoni A, et al Next-generation sequencing reveals DGUOK mutations in adult patients with mitochondrial DNA multiple deletions. Brain. 2012;135(Pt 11):3404–3415. 23043144 10.1093/brain/aws258 PMC3501975

[9] Garone C, Garcia-Diaz B, Emmanuele V, et al Deoxypyrimidine monophosphate bypass therapy for thymidine kinase 2 deficiency. EMBO Mol Med. 2014;6(8):1016–1027. 24968719 10.15252/emmm.201404092 PMC4154130

[10] Munro B, Horvath R, Muller JS. Nucleoside supplementation modulates mitochondrial DNA copy number in the dguok -/- zebrafish. Hum Mol Genet. 2019;28(5):796–803. 30428046 10.1093/hmg/ddy389 PMC6381312

[11] Caporali L, Bello L, Tagliavini F, et al DGUOK recessive mutations in patients with CPEO, mitochondrial myopathy, parkinsonism and mtDNA deletions. Brain. 2018;141(1):e3. 29228108 10.1093/brain/awx301

[12] Dogulu N, Tuna Kirsaclioglu C, Kose E, et al The clinical variations and diagnostic challenges of deoxyguanosine kinase deficiency: A descriptive case series. J Pediatr Endocrinol Metab. 2021;34(10):1341–1347. 34167177 10.1515/jpem-2021-0108

[13] Nobre S, Grazina M, Silva F, Pinto C, Goncalves I, Diogo L. Neonatal liver failure due to deoxyguanosine kinase deficiency. BMJ Case Rep. 2012;2012:bcr1220115317. 10.1136/bcr.12.2011.5317 PMC3339167 22602837

[14] Pronicka E, Weglewska-Jurkiewicz A, Taybert J, et al Post mortem identification of deoxyguanosine kinase (DGUOK) gene mutations combined with impaired glucose homeostasis and iron overload features in four infants with severe progressive liver failure. J Appl Genet. 2011;52(1):61–66. 21107780 10.1007/s13353-010-0008-y PMC3026684

[15] Hanchard NA, Shchelochkov OA, Roy A, et al Deoxyguanosine kinase deficiency presenting as neonatal hemochromatosis. Mol Genet Metab. 2011;103(3):262–267. 21478040 10.1016/j.ymgme.2011.03.006

[16] Salviati L, Sacconi S, Mancuso M, et al Mitochondrial DNA depletion and dGK gene mutations. Ann Neurol. 2002;52(3):311–317. 12205643 10.1002/ana.10284

[17] Brahimi N, Jambou M, Sarzi E, et al The first founder DGUOK mutation associated with hepatocerebral mitochondrial DNA depletion syndrome. Mol Genet Metab. 2009;97(3):221–226. 19394258 10.1016/j.ymgme.2009.03.007

[18] Buchaklian AH, Helbling D, Ware SM, Dimmock DP. Recessive deoxyguanosine kinase deficiency causes juvenile onset mitochondrial myopathy. Mol Genet Metab. 2012;107(1–2):92–94. 22622127 10.1016/j.ymgme.2012.04.019

[19] Bychkov IO, Itkis YS, Tsygankova PG, et al Mitochondrial DNA maintenance disorders in 102 patients from different parts of Russia: Mutational spectrum and phenotypes. Mitochondrion. 2021;57:205–212. 33486010 10.1016/j.mito.2021.01.004

[20] Fang W, Song P, Xie X, et al A fatal case of mitochondrial DNA depletion syndrome with novel compound heterozygous variants in the deoxyguanosine kinase gene. Oncotarget. 2017;8(48):84309–84319. 29137425 10.18632/oncotarget.20905 PMC5663597

[21] Grabhorn E, Tsiakas K, Herden U, et al Long-term outcomes after liver transplantation for deoxyguanosine kinase deficiency: A single-center experience and a review of the literature. Liver Transpl. 2014;20(4):464–472. 24478274 10.1002/lt.23830

[22] Hassan S, Mahmoud A, Mohammed TO, Mohammad S. Pediatric liver transplantation from a living donor in mitochondrial disease: Good outcomes in DGUOK deficiency? Pediatr Transplant. 2020;24(4):e13714. 32320107 10.1111/petr.13714

[23] Haudry C, de Lonlay P, Malan V, et al Maternal uniparental disomy of chromosome 2 in a patient with a DGUOK mutation associated with hepatocerebral mitochondrial DNA depletion syndrome. Mol Genet Metab. 2012;107(4):700–704. 23141463 10.1016/j.ymgme.2012.10.008

[24] Jankowska I, Czubkowski P, Rokicki D, et al Acute liver failure due to DGUOK deficiency-is liver transplantation justified? Clin Res Hepatol Gastroenterol. 2021;45(1):101408. 32278775 10.1016/j.clinre.2020.02.018

[25] Ji JQ, Dimmock D, Tang LY, et al A novel c.592-4_c.592-3delTT mutation in DGUOK gene causes exon skipping. Mitochondrion. 2010;10(2):188–191. 19900589 10.1016/j.mito.2009.11.002

[26] Majdalani M, Yazbeck N, El Harake L, Samaha J, Karam PE. Mitochondrial depletion syndrome type 3: The Lebanese variant. Front Genet. 2023;14:1215083. 37456661 10.3389/fgene.2023.1215083 PMC10339285

[27] Kasapkara CS, Tumer L, Kucukcongar A, Hasanoglu A, Seneca S, De Meirleir L. DGUOK-related mitochondrial DNA depletion syndrome in a child with an early diagnosis of glycogen storage disease. J Pediatr Gastroenterol Nutr. 2013;57(5):e28–e29. 22868686 10.1097/MPG.0b013e31826bd4ed

[28] Labarthe F, Dobbelaere D, Devisme L, et al Clinical, biochemical and morphological features of hepatocerebral syndrome with mitochondrial DNA depletion due to deoxyguanosine kinase deficiency. J Hepatol. 2005;43(2):333–341. 15964659 10.1016/j.jhep.2005.03.023

[29] Mahjoub G, Habibzadeh P, Dastsooz H, et al Clinical and molecular characterization of three patients with hepatocerebral form of mitochondrial DNA depletion syndrome: A case series. BMC Med Genet. 2019;20(1):167. 31664948 10.1186/s12881-019-0893-9 PMC6819644

[30] Montano V, Simoncini C, Calì CL, Legati A, Siciliano G, Mancuso M. CPEO and mitochondrial myopathy in a patient with DGUOK compound heterozygous pathogenetic variant and mtDNA multiple deletions. Case Rep Neurol Med. 2019;2019:5918632. 30956829 10.1155/2019/5918632 PMC6431376

[31] Navarro-Sastre A, Tort F, Garcia-Villoria J, et al Mitochondrial DNA depletion syndrome: New descriptions and the use of citrate synthase as a helpful tool to better characterise the patients. Mol Genet Metab. 2012;107(3):409–415. 22980518 10.1016/j.ymgme.2012.08.018

[32] AlMenabawy N, Hassaan HM, Ramadan M, et al Clinical and genetic spectrum of mitochondrial DNA depletion syndromes: A report of 6 cases with 4 novel variants. Mitochondrion. 2022;65:139–144. 35750291 10.1016/j.mito.2022.06.004

[33] Sezer T, Ozcay F, Balci O, Alehan F. Novel deoxyguanosine kinase gene mutations in the hepatocerebral form of mitochondrial DNA depletion syndrome. J Child Neurol. 2015;30(1):124–128. 24423689 10.1177/0883073813517000

[34] Slama A, Giurgea I, Debrey D, et al Deoxyguanosine kinase mutations and combined deficiencies of the mitochondrial respiratory chain in patients with hepatic involvement. Mol Genet Metab. 2005;86(4):462–465. 16263314 10.1016/j.ymgme.2005.09.006

[35] McKiernan P, Ball S, Santra S, et al Incidence of primary mitochondrial disease in children younger than 2 years presenting with acute liver failure. J Pediatr Gastroenterol Nutr. 2016;63(6):592–597. 27482763 10.1097/MPG.0000000000001345 PMC5113754

[36] Unal O, Hismi B, Kilic M, et al Deoxyguanosine kinase deficiency: A report of four patients. J Pediatr Endocrinol Metab. 2017;30(6):697–702. 28493820 10.1515/jpem-2016-0268

[37] Vilarinho S, Sari S, Yilmaz G, et al Recurrent recessive mutation in deoxyguanosine kinase causes idiopathic noncirrhotic portal hypertension. Hepatology. 2016;63(6):1977–1986. 26874653 10.1002/hep.28499 PMC4874872

[38] Waich S, Roscher A, Brunner-Krainz M, et al Severe deoxyguanosine kinase deficiency in Austria: A 6-patient series. J Pediatr Gastroenterol Nutr. 2019;68(1):e1–e6. 30589726 10.1097/MPG.0000000000002149

[39] Mousson de Camaret B, Taanman JW, Padet S, et al Kinetic properties of mutant deoxyguanosine kinase in a case of reversible hepatic mtDNA depletion. Biochem J. 2007;402(2):377–385. 17073823 10.1042/BJ20060705 PMC1798436

[40] Tadiboyina VT, Rupar A, Atkison P, et al Novel mutation in DGUOK in hepatocerebral mitochondrial DNA depletion syndrome associated with cystathioninuria. Am J Med Genet A. 2005;135(3):289–291. 15887277 10.1002/ajmg.a.30748

[41] Rabinowitz SS, Gelfond D, Chen CK, et al Hepatocerebral mitochondrial DNA depletion syndrome: Clinical and morphologic features of a nuclear gene mutation. J Pediatr Gastroenterol Nutr. 2004;38(2):216–220. 14734888 10.1097/00005176-200402000-00022

[42] Wang L, Limongelli A, Vila MR, Carrara F, Zeviani M, Eriksson S. Molecular insight into mitochondrial DNA depletion syndrome in two patients with novel mutations in the deoxyguanosine kinase and thymidine kinase 2 genes. Mol Genet Metab. 2005;84(1):75–82. 15639197 10.1016/j.ymgme.2004.09.005

[43] De Greef E, Christodoulou J, Alexander IE, et al Mitochondrial respiratory chain hepatopathies: Role of liver transplantation. A case series of five patients. JIMD Rep. 2012;4:5–11. 23430890 10.1007/8904_2011_29 PMC3509872

[44] Muller-Hocker J, Muntau A, Schafer S, et al Depletion of mitochondrial DNA in the liver of an infant with neonatal giant cell hepatitis. Hum Pathol. 2002;33(2):247–253. 11957153 10.1053/hupa.2002.31477

[45] Freisinger P, Futterer N, Lankes E, et al Hepatocerebral mitochondrial DNA depletion syndrome caused by deoxyguanosine kinase (DGUOK) mutations. Arch Neurol. 2006;63(8):1129–1134. 16908739 10.1001/archneur.63.8.1129

[46] Mancuso M, Ferraris S, Pancrudo J, et al New DGK gene mutations in the hepatocerebral form of mitochondrial DNA depletion syndrome. Arch Neurol. 2005;62(5):745–747. 15883261 10.1001/archneur.62.5.745

[47] Mancuso M, Filosto M, Tsujino S, et al Muscle glycogenosis and mitochondrial hepatopathy in an infant with mutations in both the myophosphorylase and deoxyguanosine kinase genes. Arch Neurol. 2003;60(10):1445–1447. 14568816 10.1001/archneur.60.10.1445

[48] Douglas GV, Wiszniewska J, Lipson MH, et al Detection of uniparental isodisomy in autosomal recessive mitochondrial DNA depletion syndrome by high-density SNP array analysis. J Hum Genet. 2011;56(12):834–839. 22011815 10.1038/jhg.2011.112 PMC7512120

[49] Lee NC, Dimmock D, Hwu WL, et al Simultaneous detection of mitochondrial DNA depletion and single-exon deletion in the deoxyguanosine gene using array-based comparative genomic hybridisation. Arch Dis Child. 2009;94(1):55–58. 19103789 10.1136/adc.2008.139584

[50] Kilic M, Sivri HS, Dursun A, et al A novel mutation in the DGUOK gene in a Turkish newborn with mitochondrial depletion syndrome. Turk J Pediatr. 2011;53(1):79–82. 21534344

[51] Mudd SH, Wagner C, Luka Z, et al Two patients with hepatic mtDNA depletion syndromes and marked elevations of S-adenosylmethionine and methionine. Mol Genet Metab. 2012;105(2):228–236. 22137549 10.1016/j.ymgme.2011.11.006 PMC3264801

[52] Shieh JT, Berquist WE, Zhang Q, Chou PC, Wong LJ, Enns GM. Novel deoxyguanosine kinase gene mutations and viral infection predispose apparently healthy children to fulminant liver failure. J Pediatr Gastroenterol Nutr. 2009;49(1):130–132. 19502998 10.1097/MPG.0b013e31819de7a6

[53] Arya VB, Dhawan A, Kapoor RR. Hyperinsulinaemic hypoglycaemia in deoxyguanosine kinase deficiency. Clin Endocrinol (Oxf). 2019;91(6):900–903. 31465631 10.1111/cen.14084

[54] Filosto M, Mancuso M, Tomelleri G, et al Hepato-cerebral syndrome: Genetic and pathological studies in an infant with a dGK mutation. Acta Neuropathol. 2004;108(2):168–171. 15150663 10.1007/s00401-004-0872-9

[55] Xia JK, Bai ZX, Zhao XC, Meng JJ, Chen C, Kong XD. Mitochondrial DNA depletion syndrome in a newborn with jaundice caused by DGUOK mutation and complete uniparental disomy of chromosome 2. Pediatr Neonatol. 2020;61(5):558–560. 32482602 10.1016/j.pedneo.2020.04.005

[56] Yamazaki T, Murayama K, Compton AG, et al Molecular diagnosis of mitochondrial respiratory chain disorders in Japan: Focusing on mitochondrial DNA depletion syndrome. Pediatr Int. 2014;56(2):180–187. 24266892 10.1111/ped.12249

[57] Lipinski P, Ciara E, Jurkiewicz D, et al Targeted next-generation sequencing in diagnostic approach to monogenic cholestatic liver disorders-single-center experience. Front Pediatr. 2020;8:414. 32793533 10.3389/fped.2020.00414 PMC7393978

[58] Komal FNU, Moretti PM, Shaibani AI. Deoxyguanosine kinase mutation producing juvenile-onset mitochondrial myopathy. Neurol Genet. 2018;4(5):e269. 30283818 10.1212/NXG.0000000000000269 PMC6167180

[59] Alberio S, Mineri R, Tiranti V, Zeviani M. Depletion of mtDNA: Syndromes and genes. Mitochondrion. 2007;7(1–2):6–12. 17280874 10.1016/j.mito.2006.11.010

[60] Calvo SE, Compton AG, Hershman SG, et al Molecular diagnosis of infantile mitochondrial disease with targeted next-generation sequencing. Sci Transl Med. 2012;4(118):118ra10. 10.1126/scitranslmed.3003310 PMC3523805 22277967

[61] Taylor RW, Pyle A, Griffin H, et al Use of whole-exome sequencing to determine the genetic basis of multiple mitochondrial respiratory chain complex deficiencies. JAMA. 2014;312(1):68–77. 25058219 10.1001/jama.2014.7184 PMC6558267

[62] Zeviani M, Gellera C, Pannacci M, et al Tissue distribution and transmission of mitochondrial DNA deletions in mitochondrial myopathies. Ann Neurol. 1990;28(1):94–97. 2375642 10.1002/ana.410280118

[63] Spinazzola A, Viscomi C, Fernandez-Vizarra E, et al MPV17 encodes an inner mitochondrial membrane protein and is mutated in infantile hepatic mitochondrial DNA depletion. Nat Genet. 2006;38(5):570–575. 16582910 10.1038/ng1765

[64] Almannai M, El-Hattab AW, Azamian MS, Ali M, Scaglia F. Mitochondrial DNA maintenance defects: Potential therapeutic strategies. Mol Genet Metab. 2022;137(1–2):40–48. 35914366 10.1016/j.ymgme.2022.07.003 PMC10401187

[65] Almannai M, Salah A, El-Hattab AW. Mitochondrial membranes and mitochondrial genome: Interactions and clinical syndromes. Membranes (Basel). 2022;12(6):625. 35736332 10.3390/membranes12060625 PMC9229594

[66] Lam CW, Yeung WL, Ling TK, Wong KC, Law CY. Deoxythymidylate kinase, DTYMK, is a novel gene for mitochondrial DNA depletion syndrome. Clin Chim Acta. 2019;496:93–99. 31271740 10.1016/j.cca.2019.06.028

[67] Kornblum C, Nicholls TJ, Haack TB, et al Loss-of-function mutations in MGME1 impair mtDNA replication and cause multisystemic mitochondrial disease. Nat Genet. 2013;45(2):214–219. 23313956 10.1038/ng.2501 PMC3678843

[68] Harel T, Yoon WH, Garone C, et al Recurrent de novo and biallelic variation of ATAD3A, encoding a mitochondrial membrane protein, results in distinct neurological syndromes. Am J Hum Genet. 2016;99(4):831–845. 27640307 10.1016/j.ajhg.2016.08.007 PMC5065660

[69] Garone C, Tadesse S, Hirano M. Clinical and genetic spectrum of mitochondrial neurogastrointestinal encephalomyopathy. Brain. 2011;134(Pt 11):3326–3332. 21933806 10.1093/brain/awr245 PMC3212717

[70] Garone C, Taylor RW, Nascimento A, et al Retrospective natural history of thymidine kinase 2 deficiency. J Med Genet. 2018;55(8):515–521. 29602790 10.1136/jmedgenet-2017-105012 PMC6073909

[71] Almannai M, El-Hattab AW, Scaglia F. Mitochondrial DNA replication: Clinical syndromes. Essays Biochem. 2018;62(3):297–308. 29950321 10.1042/EBC20170101

[72] Bliksrud YT, Brodtkorb E, Andresen PA, van den Berg IE, Kvittingen EA. Tyrosinaemia type I–de novo mutation in liver tissue suppressing an inborn splicing defect. J Mol Med (Berl). 2005;83(5):406–410. 15759101 10.1007/s00109-005-0648-2

[73] Dominguez-Gonzalez C, Hernandez-Lain A, Rivas E, et al Late-onset thymidine kinase 2 deficiency: A review of 18 cases. Orphanet J Rare Dis. 2019;14(1):100. 31060578 10.1186/s13023-019-1071-z PMC6501326

[74] Eldredge JA, Hardikar W. Current status and future directions of liver transplantation for metabolic liver disease in children. Pediatr Transplant. 2023;28:e14625. 37859572 10.1111/petr.14625

[75] Dimmock DP, Dunn JK, Feigenbaum A, et al Abnormal neurological features predict poor survival and should preclude liver transplantation in patients with deoxyguanosine kinase deficiency. Liver Transpl. 2008;14(10):1480–1485. 18825706 10.1002/lt.21556

[76] Dominguez-Gonzalez C, Madruga-Garrido M, Mavillard F, et al Deoxynucleoside therapy for thymidine kinase 2-deficient myopathy. Ann Neurol. 2019;86(2):293–303. 31125140 10.1002/ana.25506 PMC7586249

