# Supplementary material for: Deoxyguanosine kinase deficiency: natural history and liver transplant outcome
Source: Brain Commun. 2024 May 6;6(3):fcae160. doi: 10.1093/braincomms/fcae160 (PMC11098040; doi:10.1093/braincomms/fcae160)
Supplement: fcae160_Supplementary_Data [file fcae160_supplementary_data.zip › Supplementary_Figures.pdf]

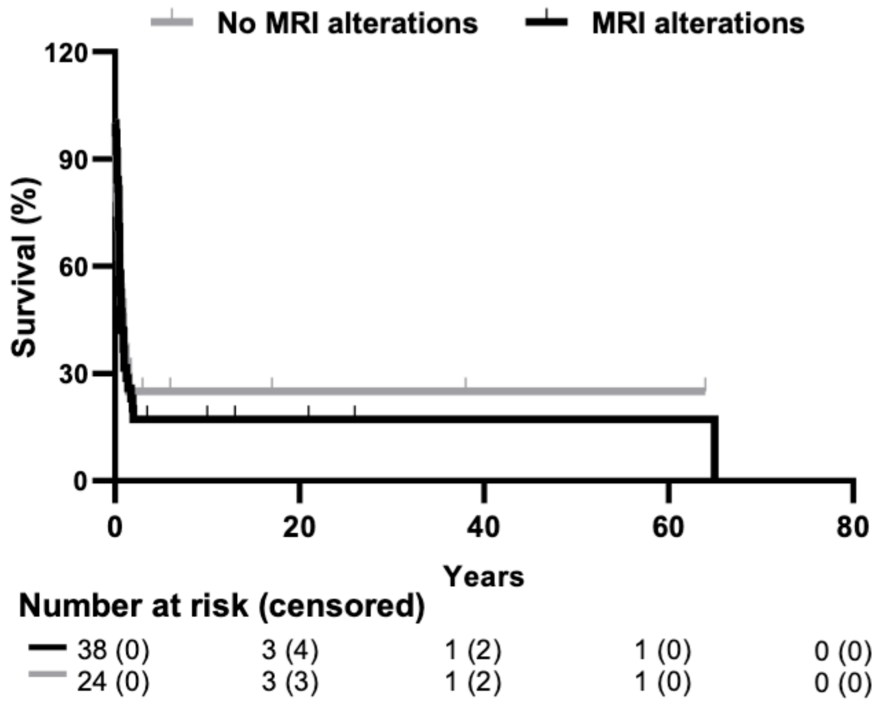

Supplementary Figure 1: Kaplan-Meier curve comparing survival rate in the subgroups of patients with or without Brain MRI abnormalities (p-value =0.8450; n=62). Survival analysis was assessed by log-rank Mantel-Cox test. Numbers at risk are specified below each panel. When statistically significant, p values are indicated in the figure. Abbreviations: MRI: Magnetic Resonance Imaging.

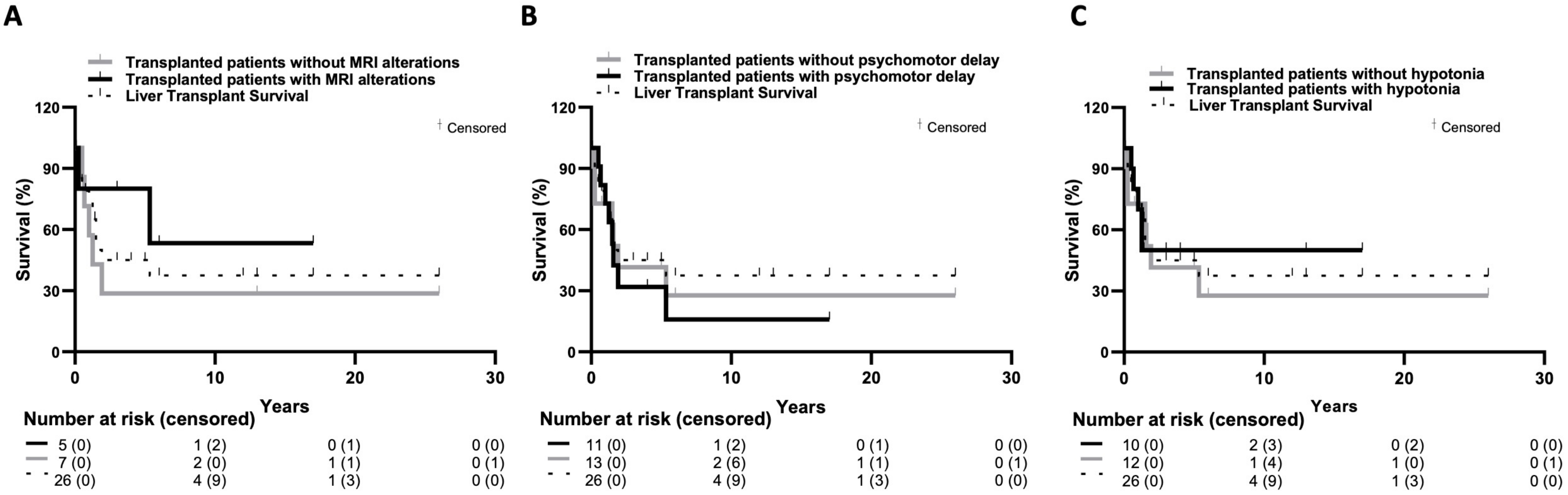

Supplementary Figure 2: Kaplan-Meier analysis of LTx prognostic factors: Survival rate in subgroups of LTx patients (n=26) with or without MRI alterations (p-value =0.3196, n=12) (A), psychomotor delay (p-value =0.7040, n=24) (B) or central hypotonia (p-value =0.6793, n=22) (C). Survival analysis was assessed by log-rank Mantel-Cox test. Numbers at risk are specified below each panel. When statistically significant, p values are indicated in the figure. Abbreviations: LTx: liver transplant; MRI: Magnetic Resonance Imaging.
