# Supplementary material for: Deoxyguanosine kinase deficiency: natural history and liver transplant outcome
Source: Brain Commun. 2024 May 6;6(3):fcae160. doi: 10.1093/braincomms/fcae160 (PMC11098040; doi:10.1093/braincomms/fcae160)
Supplement: fcae160_Supplementary_Data [file fcae160_supplementary_data.zip › Supplementary_Tables.pdf]

Supplementary Table 1: Principal features of the patient's cohort.

					Laboratory Tests								
**P#**	**Sex**	**Onset (m)**	**Age of onset group**	**Clinical phenotype**	**CK**	**Transaminases**	**Plasma amino acids**	**mtDNA multiple deletions**	**DGUOK mutations**	**Liver Transplant**	**Death/Age (y)**	**Cause of Death**	**Ref.**
1	F	240,0	Adult	Myopathy	↑437 U/L	- / -	NA	+	p.Asn154Lys; p.Gln192*	-	+/65	Non-Hodgkin's lymphoma	UP
2	F	468,0	Adult	Myopathy	-	- / -	NA	+	p.Gln192*	-	-/58	-	UP
3	F	480,0	Adult	Myopathy	↑243 U/L	- / -	NA	+	p.Asn154Lys; c.707+2T>G	-	-/40	-	30
4	M	504,0	Adult	Myopathy	↑ 450 U/L	↑ 90 / ↑ 88	NA	+	p.Asn154Lys	-	-/58	-	UP
5	F	408,0	Adult	Myopathy	-	- / -	NA	NA	p.Arg39Gly; p.Phe256*	-	-/55	-	UP
6	M	420,0	Adult	Myopathy	↑ 346 U/L	- / -	NA	NA	p.Arg39Gly; p.Phe256*	-	-/53	-	UP
7	F	300,0	Adult	Myopathy	↑ 1094 U/L	↑ 44/ ↑ 54	NA	NA	p.Arg39Gly; p.Phe256*	-	-/56	-	UP
8	M	0,033	Neonatal	Hepatomyo cerebral	NA	NA	↑ Tyr	NA	p.Arg105*;	-	+/0.5	Liver failure	UP
9	M	420,0	Adult	Myopathy	-	- / -	NA	NA	p.Met1Ile; p.Pro196Ser	-	-/64	-	11
10	F	360,0	Adult	Myopathy	↑ 397 U/L	- / -	NA	+	p.Met1Ile; p.Pro196Ser	-	-/64	-	11
11	F	600,0	Adult	Myopathy	↑ 270 U/L	- / -	NA	+	p.Glu44Lys; p.Thr235Arg	-	NA	NA	UP
12	F	0,033	Neonatal	Hepatomyo cerebral	-	↑ 61 / ↑ 176	↑ Tyr, Ala	NA	p.Glu44Lys; p.Arg142Valfs*3	-	+/0.5	Liver failure	UP
13	F	0,1	Neonatal	Hepatocerebral	NA	↑ / ↑	NA	-	p.Phe256*	-	+/0.3	NA	28

14	M	0,5	Neonatal	Hepatocerebral	NA	↑ / ↑	NA	NA	p.Phe256*	-	+/0.6	NA	28
15	M	0,5	Neonatal	Hepatocerebral	NA	↑ / ↑	NA	-	p.Phe256*	-	+/1.8	NA	28
16	M	0,033	Neonatal	Hepatocerebral	NA	NA	NA	NA	p.Phe256*	-	+/0.1	NA	28
17	M	0,033	Neonatal	Hepatocerebral	NA	↑ / ↑	NA	-	p.Phe256*	-	+/0.3	NA	28
18	F	0,033	Neonatal	Hepatocerebral	↑ 464 U/L	↑ / ↑	NA	-	p.Phe256*	-	+/0.2	Liver failure	28
19	F	0,2	Neonatal	Hepatomyo cerebral	NA	↑ 159 / ↑ 250	-	NA	p.Phe256*;	-	+/0.4	Liver failure	41
20	F	0,033	Neonatal	Hepatomyo cerebral	NA	↑ 83 / ↑ 163	-	-	p.Phe256*;	-	+/0.5	Liver failure, GI bleeding	41
21	M	0,3	Neonatal	Hepatopathy	NA	↑ / ↑	-	NA	p.Phe256*;	+	+/0.3	MOF	41
22	F	2,0	Infantile	Hepatocerebral	NA	↑159 / ↑250	NA	NA	p.Phe256*;	NA	+/0.4	Shock	16
23	M	0,2	Neonatal	Hepatocerebral	NA	↑157 / ↑425	NA	NA	p.Tyr204ProfsTer 11;	-	+/0.5	Liver failure	16
24	M	6,0	Infantile	Hepatopathy	NA	↑88 / ↑200	NA	NA	p.Arg142Lys; p.Glu227Lys	+	-/5	-	16
25	M	2,0	Infantile	Hepatocerebral	NA	↑ / ↑	NA	NA	p.Asp255Tyr	-	+/0.7	Liver failure	40
26	F	2,0	Infantile	Hepatocerebral	NA	↑ / ↑	NA	NA	p.Asp255Tyr	-	+/0.7	Liver failure	40
27	F	2,0	Infantile	Hepatocerebral	NA	↑ / ↑	NA	NA	p.Asp255Tyr	+	-/4	-	40
28	F	0,033	Neonatal	Hepatocerebral	NA	NA	NA	NA	p.Leu250Ser;	-	+/2	NA	42
29	M	168,0	Paediatric	Myopathy	NA	NA	-	NA	p.Ala2Ser; p.Pro28Serter57	-	-/21	-	18

30	M	0,033	Neonatal	Hepatocerebral	-	↑655 / ↑151	↑ Ala	NA	p.His226Arg;	-	+/0.4	MOF	13
31	F	0,1	Neonatal	Hepatocerebral	-	↑198 / ↑95	↑ Tyr	NA	p.His226Arg;	-	+/0.2	NA	13
32	M	0,1	Neonatal	Hepatocerebral	-	↑359 / ↑237	↑ Tyr	NA	p.Leu250Ser;	+	-/1.4	-	13
33	M	0,1	Neonatal	Hepatocerebral	NA	↑82 / ↑195	↑ Tyr	NA	p.Glu44Lys	-	+/0.5	MOF	38
34	F	0,033	Neonatal	Hepatocerebral	NA	↑215 / ↑536	↑ Tyr	NA	p.Glu44Lys	-	+/0.9	MOF	38
35	M	0,033	Neonatal	Hepatopathy	NA	↑536 / ↑844	↑ Tyr	NA	p.Arg105*	-	+/0.2	MOF	38
36	F	1,0	Neonatal	Hepatopathy	NA	↑151 / ↑269	↑ Tyr	NA	p.Ala48Glyfs43; p.Gln221Pro	+	-/0.1	-	38
37	F	0,033	Neonatal	Hepatopathy	NA	↑115 / ↑310	NA	NA	p.Ala48Glyfs43	-	+/0.1	MOF	38
38	F	0,1	Neonatal	Hepatocerebral	NA	NA / ↑ 115	↑ Tyr	NA	p.Ala48Glyfs43; p.Leu192Pro	-	+/0.3	MOF, hypertensive crisis	38
39	F	696,0	Adult	Myopathy	↑	- / -	NA	+	p.Asn154Lys; p.Arg202TyrfsX12	-	NA	NA	8
40	F	552,0	Adult	Myopathy	↑ 2000 U/L	- / -	NA	+	p.Glu44Lys; p.Asn154Lys	-	-/46	-	8
41	M	828,0	Adult	Myopathy	↑ 257 U/L	↑34 / ↑139	NA	+	p.Tyr62X; p.Gln170Arg	-	+/69	NA	8
42	F	9,0	Infantile	Hepatopathy	↑ 13000 U/L	NA / ↑800	NA	+	p.Asn46Ser; p.Arg202TyrfsX12	+	-/0.8	-	8
43	F	480,0	Adult	Myopathy	↑ 196 U/L	- / -	NA	+	c.444-11C>G; p.Gln170Arg	-	NA	NA	8
44	M	528,0	Adult	Myopathy	↑ 235 U/L	- / -	NA	+	c.444-11C>G; p.Gln170Arg	-	NA	NA	8
45	M	7,0	Infantile	Hepatopathy	-	↑154 / ↑630	NA	-	putative splicing mutation	+	+/0.2	Liver failure	43

46	M	0,1	Neonatal	Hepatocerebral	-	NA	↑ Tyr, Gln, Ala	-	p.Arg105*;	-	+/0.4	Liver failure	45
47	F	0,033	Neonatal	Hepatocerebral	NA	NA	NA	NA	p.Met1Thr;	-	+/0.3	Liver failure	34
48	M		-	Hepatocerebral	NA	NA	NA	NA	p.Phe256*;	-	+/0.3	Liver failure	34
49	F		-	Hepatocerebral	NA	NA	NA	NA	p.Leu250Ser;	-	+	Liver failure	34
50	M	4,0	Infantile	Hepatopathy	NA	↑430 / NA	↑ Tyr, Met, Phe	NA	p.Glu211Gly; p.Leu266Arg	-	+/0.5	MOF	34
51	M		-	Hepatocerebral	NA	NA	NA	NA	p.Glu165Val; p.Leu266Arg	+	+/1.5	Pulmonary arterial hypertension	34
52	M	2,0	Infantile	Hepatopathy	-	↑104 / ↑134	NA	-	p.Asn46Ser; p.Leu266Arg	-	-/10	-	39
53	M	0,033	Neonatal	Hepatopathy	NA	NA	NA	-	p.Ala2Ser; p.Gln197=; c.142+1G>A	-	+/0.3	Liver failure, encephalopath y	39
54	F	0,033	Neonatal	Hepatopathy	NA	NA	NA	-	p.Phe256*	-	+/0.2	Liver failure	39
55	F	8,0	Infantile	Hepatocerebral	NA	↑573 / ↑1207	NA	-	p.Trp75Arg;	-	+/0.9	Liver failure	7
56	M	1,0	Neonatal	Hepatocerebral	NA	↑182 / ↑309	↑ Tyr, Ala, Met	-	p.Phe256*;	-	+/0.7	Liver failure	7
57	M	3,0	Infantile	Hepatocerebral	NA	- / ↑45	NA	-	p.Phe256*;	-	+/0.8	Liver failure	7
58	F	1,0	Neonatal	Hepatocerebral	NA	↑268 / ↑63	NA	-	p.Arg206Lys	-	NA	NA	7
59	F	0,2	Neonatal	Hepatomyo cerebral	-	↑60 / ↑92	-	NA	p.Asp255Tyr	+	-/13	-	21
60	F	2,0	Infantile	Hepatocerebral	-	↑ / ↑	↑ Tyr	-	p.Met1Val; p.Met1Ile	+	-/17	-	21
61	M	1,5	Infantile	Hepatomyocere bral	NA	↑ / ↑	NA	-	p.Ser52Phe	-	-/1	-	45

62	F	0,5	Neonatal	Hepatocerebral	NA	NA	↑ Tyr	NA	p.Ser52Phe; c.681-684delGTTT p.Glu227AspfsTer15	-	+/1.1	Liver failure	45
63	M	0,1	Neonatal	Hepatocerebral	NA	NA	NA	NA	p.Met1Thr; p.Leu250Ser	-	+/0.8	Liver failure	45
64	F	0,1	Neonatal	Hepatopathy	NA	↑ / ↑	NA	-	p.Met1Thr; p.Leu250Ser	-	-/0.4	-	45
65	F	3,0	Infantile	Hepatocerebral	NA	NA	NA	-	p.Gln170Arg	-	-/21	-	45
66	F	0,033	Neonatal	Hepatopathy	NA	- / -	↑ Tyr, Met, Ala	-	p.Tyr191Cys;	-	+/0.1	Liver failure	15
67	-	0,3	Neonatal	Hepatopathy	NA	NA	NA	-	p.Glu44Lys	-	+/0.1	Liver failure	35
68	-	0,5	Neonatal	Hepatopathy	NA	NA	NA	-	p.Arg39X	-	+/0.1	Liver failure	35
69	M	2,0	Infantile	Hepatocerebral	-	↑123 / ↑270	-	-	p.Glu44Lys	-	+/0.5	Liver failure	33
70	F	3,0	Infantile	Hepatocerebral	-	↑293 / ↑785	-	-	p.Glu165Lys	-	+/0.9	Liver failure	33
71	F	2,0	Infantile	Hepatocerebral	-	↑87 / ↑246	-	-	c.707+3_6delTAAG	-	+/0.6	Liver failure	33
72	M	144,0	Paediatric	Hepatopathy	NA	- / -	NA	NA	p.Asn46Ser	-	-/19	-	37
73	M	5,0	Infantile	Hepatopathy	NA	↑105 / ↑293	NA	NA	p.Asn46Ser	-	-/17	-	37
74	F	60,0	Paediatric	Hepatopathy	NA	- / -	NA	NA	p.Asn46Ser	-	-/12	-	37
75	M	7,0	Infantile	Hepatopathy	NA	↑101 / ↑136	NA	NA	p.Arg118His; p.Glu227Lys	+	-/6	-	22
76	F	0,033	Neonatal	Hepatocerebral	NA	↑ / ↑	NA	NA	c.269C>T;	-	+/0.4	Encephalopathy	46

77	F	6,0	Infantile	Hepatocerebral	NA	NA	NA	NA	c.269C>T;	-	+/0.3	Liver failure	46
78	F	0,033	Neonatal	Hepatocerebral	NA	↑73 / ↑163	NA	NA	c.352G>A;	+	+/0.3	Pulmonary arterial hypertension	46
79	F	0,033	Neonatal	Hepatocerebral	↑	NA	NA	NA	p.Phe256*	-	+/0.4	Liver failure	47
80	F	1,0	Neonatal	Hepatocerebral	NA	↑ / ↑	↑ Tyr	NA	c.80delC; p.Phe256*	+	+/0.5	Peritonitis	4
81	M	3,0	Infantile	Hepatocerebral	NA	↑175 / NA	↑ Tyr	NA	p.Trp65X; c.487_490dupGACA p.Ile164ArgfsTer53	-	+/1	Liver failure	4
82	M	2,0	Infantile	Hepatocerebral	NA	NA	NA	-	p.Trp65X; c.487_490dupGACA p.Ile164ArgfsTer53	-	+/0.6	Liver failure	4
83	M	1,6	Infantile	Hepatocerebral	NA	↑ / ↑	↑ Tyr, Met, Phe	-	p.Gln197=; p.Arg202TyrfsX12	+	+/1.9	Cardiac arrest	4
84	F	3,0	Infantile	Hepatocerebral	NA	↑ / ↑	↑ Tyr	-	p.Trp178X	+	+/1.3	LT complications	4
85	F	0,033	Neonatal	Hepatocerebral	NA	NA	↑ Tyr	-	p.Lys51Gln; p.Tyr191Cys	-	+/0.7	Liver failure	4
86	M	10,0	Infantile	Hepatopathy	NA	↑ / ↑	NA	-	p.Asn46Ser; p.Arg118Cys	-	+/0.8	Cardiopulmonary arrest	4
87	M	10,0	Infantile	Hepatopathy	NA	↑ / ↑	NA	-	p.Asn46Ser; p.Arg118Cys	-	-/3.5	-	4
88	M	0,033	Neonatal	Hepatocerebral	NA	NA	-	-	p.His226Arg; c.592-4_592-3delTT	-	+/0.7	Liver failure	4
89	M	0,033	Neonatal	Hepatocerebral	NA	↑ / ↑	↑ Tyr, Met	-	p.Arg202TyrfsX12	-	+/0.6	Liver failure	4
90	M	6,0	Infantile	Hepatopathy	NA	↑1770 / ↑3760	NA	-	p.Ser107Pro	-	-/3.5	-	4

91	F	4,0	Infantile	Hepatocerebral	NA	NA	NA	-	c.592-4_592-3delTT;	-	+/0.5	Liver failure	25
92	M	1,4	Infantile	Hepatocerebral	NA	↑59 / ↑132	↑ Tyr, Met, Ala	-	c.592-4_592-3delTT;	-	-/0.1	-	25
93	F	0,2	Neonatal	Hepatocerebral	NA	↑ / ↑	↑ Tyr	NA	p.Trp178X	+	+/1	Liver failure	48
94	M	0,1	Neonatal	Hepatocerebral	NA	↑58 / ↑119	↑ Tyr, Ala, Gln	NA	p.Glu227Lys	-	-/0.3	-	49
95	M	0,1	Neonatal	Hepatocerebral	NA	↑46 / ↑180	↑ Tyr, Ala	-	p.Glu227Lys	-	+/0.3	Liver failure	49
96	F	0,1	Neonatal	Hepatocerebral	NA	↑113 / ↑201	NA	-	p.Arg118Cys	-	+/0.8	Liver failure	23
97	M	4,0	Infantile	Hepatocerebral	NA	↑58 / ↑149	↑ Tyr	NA	p.Arg105*	-	+/0.3	Liver failure	27
98	M	0,033	Neonatal	Hepatocerebral	NA	↑53 / ↑203	↑ Tyr, Ala	NA	p.Arg12X	-	+/0.1	Liver failure	50
99	F	0,1	Neonatal	Hepatocerebral	NA	↑118 / ↑162	↑ Tyr, Ala	NA	p.Arg105*	-	+/0.3	MOF	12
100	M	1,5	Infantile	Hepatocerebral	NA	↑773 / ↑1467	↑ Met, Ala	NA	p.Arg118His	+	+/1.3	Pulmonary hypertension	12
101	M	0,4	Neonatal	Hepatocerebral	NA	- / -	↑ Tyr, Met, Ala	NA	p.Arg105*	NA	NA	NA	12
102	F	0,4	Neonatal	Hepatopathy	NA	↑93 / ↑120	↑ Tyr, Met, Ala	NA	p.Glu44Lys	-	+/0.3	Liver failure	12
103	M	0,033	Neonatal	Hepatocerebral	NA	↑103 / ↑230	-	NA	p.Glu44Lys	-	+/0.5	Liver failure	36
104	M	1,7	Infantile	Hepatocerebral	NA	↑115 / ↑175	↑ Tyr, Ala	NA	p.Glu227Lys	-	+/0.4	MOF	36
105	M	0,2	Neonatal	Hepatocerebral	NA	↑99 / ↑50	↑ Tyr, Ala	NA	p.Lys236Alafs*4	-	+/0.3	Liver failure	36
106	F	0,033	Neonatal	Hepatocerebral	NA	↑262 / ↑188	↑ Tyr	-	c.707+417_c.834 +3416	-	+/0.5	Liver failure	51

107	M	10,0	Infantile	Hepatopathy	NA	↑1099 / ↑8009	NA	NA	p.Asn46Ser; p.Arg118Cys	-	+/0.8	Liver failure, cardiac arrest	52
108	M	10,0	Infantile	Hepatopathy	NA	↑2211 / ↑908	NA	-	p.Asn46Ser; p.Arg118Cys	-	-/0.8	-	52
109	M	0,033	Neonatal	Hepatopathy	-	↑172 / ↑350	↑ Tyr	NA	p.Phe256*	-	+/0.7	Liver failure	53
110	M	3,0	Infantile	Hepatopathy	NA	NA / ↑75	NA	NA	p.Phe256*	-	+/2.6	GII bleeding	54
111	F	0,033	Neonatal	Hepatocerebral	NA	↑125 / ↑120	NA	NA	p.Met1Ile	-	+/0.5	Liver failure	24
112	F	0,033	Neonatal	Hepatocerebral	NA	↑155 / ↑140	NA	NA	p.Met1Val; p.Met1Ile	-	+/0.5	Liver failure	24
113	F	0,033	Neonatal	Hepatocerebral	NA	↑88 / ↑70	NA	NA	p.Met1Val; p.Met1Ile	-	+/0.2	Liver failure	24
114	M	0,033	Neonatal	Hepatocerebral	NA	↑220 / ↑336	NA	NA	p.Met1Ile	+	+/0.7	LT complications	24
115	M	0,033	Neonatal	Hepatocerebral	NA	↑67 / ↑126	NA	NA	p.Met1Val; p.Met1Ile	+	-/3.0	-	24
116	M	0,6	Neonatal	Hepatopathy	NA	- / ↑52	NA	NA	p.Glu227Lys	-	+/0.5	Liver failure	55
117	F	0,3	Neonatal	Hepatopathy	NA	↑293 / ↑623	NA	NA	p.Ile43Thr; p.Arg105*	-	+/0.8	Liver failure	20
118	F	0,033	Neonatal	Hepatopathy	NA	↑49 / ↑215	-	NA	p.Ala48fsX90	-	+/0.8	Liver failure	56
119	F	3,0	Infantile	Hepatomyo cerebral	NA	NA	↑ Tyr	NA	p.Ala48fsX90	+	+/1.6	Cardiac arrest	56
120	F	0,3	Neonatal	Hepatocerebral	NA	↑98 / ↑272	NA	NA	p.Ala48fsX90; p.Leu248Pro	+	+/1.5	Pulmonary hypertension	56
121	F	0,033	Neonatal	Hepatocerebral	NA	↑554 / ↑942	↑ Tyr	NA	p.Lys236fs	-	+/0.5	Liver failure	29
122	F		-	Hepatocerebral	NA	NA	NA	NA	p.Lys236fs	-	+/0.3	Liver failure	29

123	F	0,033	Neonatal	Hepatomyo cerebral	NA	NA	↑ Tyr	NA	p.Met1Ile; p.Asn271-Thr272insPhe	-	+/0.5	Liver failure	14
124	M	0,033	Neonatal	Hepatomyo cerebral	NA	NA	NA	NA	p.Glu165Val	-	+/0.2	Liver failure	14
125	F	0,033	Neonatal	Hepatomyo cerebral	NA	NA	↑ Met	NA	p.Met1Ile; p.Phe256*	-	+/1.5	Liver failure	14
126	F	0,033	Neonatal	Hepatomyo cerebral	↑ 586 U/L	NA	NA	NA	p.Met1Ile	-	+/0.5	Liver failure	14
127	-		-	Hepatocerebral	NA	↑ / ↑	NA	NA	p.His226Arg;	-	+/0.3	Liver failure	31
128	-		-	Hepatocerebral	NA	↑ / ↑	NA	NA	p.His226Arg;	-	+/0.3	Liver failure	31
129	-		-	Hepatocerebral	NA	↑ / ↑	NA	NA	p.His226Arg;	-	+/0.3	Liver failure	31
130	-		-	Hepatocerebral	NA	↑ / ↑	NA	NA	p.His226Arg;	-	+/0.3	Liver failure	31
131	-		-	Hepatopathy	NA	NA	NA	NA	p.Phe256*	-	+/0.3	Liver failure	31
132	M	1,0	Neonatal	Hepatopathy	NA	- / -	NA	NA	p.Met1Val; p.Met1Ile	+	-/0.1	-	57
133	F	96,0	Paediatric	Myopathy	↑ 503 U/L	NA	NA	+	p.Trp65Ter; p.Asn154Lys	-	-/38	-	58
134	-		-	-	NA	NA	NA	NA	p.Lys201fs214X	NA	NA	NA	59
135	M	3,0	Infantile	Hepatocerebral	NA	↑59 / ↑116	↑ Gly	NA	c.444-62C>A;	-	+/1.2	Liver failure	17
136	M		-	Hepatopathy	NA	NA	NA	NA	c.444-62C>A;	NA	+/0.3	Liver failure	17
137	M	3,0	Infantile	Hepatocerebral	NA	↑390 / ↑722	↑ Met	NA	c.444-62C>A;	-	+/1.5	Liver failure	17
138	F		-	Hepatocerebral	NA	NA	NA	NA	p.Ala86Profs*13	NA	+/0.1	NA	5

139	F	-	Hepatocerebral	NA	NA	NA	NA	p.Ala86Profs*13	NA	+/0.1	NA	5
140	M	-	Hepatocerebral	NA	NA	NA	NA	p.Ala86Profs*13	NA	+/0.1	NA	5
141	F	-	Hepatocerebral	NA	NA	NA	NA	p.Ala86Profs*13	NA	+/0.1	NA	5
142	M	-	Hepatocerebral	NA	NA	NA	NA	p.Ala86Profs*13	NA	+/0.1	NA	5
143	F	-	Hepatocerebral	NA	NA	NA	NA	p.Ala86Profs*13	NA	+/0.1	NA	5
144	F	-	Hepatocerebral	NA	NA	NA	NA	p.Ala86Profs*13	NA	+/0.1	NA	5
145	M	-	Hepatocerebral	NA	NA	NA	NA	p.Ala86Profs*13	NA	+/0.1	NA	5
146	F	-	Hepatocerebral	NA	NA	NA	NA	p.Ala86Profs*13	NA	+/0.1	NA	5
147	F	-	Hepatocerebral	NA	NA	NA	NA	p.Ala86Profs*13	NA	+/0.1	NA	5
148	-	-	Hepatocerebral	NA	NA	NA	NA	p.Ala86Profs*13	NA	+/0.1	NA	5
149	-	-	Hepatocerebral	NA	NA	NA	NA	p.Ala86Profs*13	NA	+/0.1	NA	5
150	-	-	Hepatocerebral	NA	NA	NA	NA	p.Ala86Profs*13	NA	+/0.1	NA	5
151	-	-	Hepatocerebral	NA	NA	NA	NA	p.Ala86Profs*13	NA	+/0.1	NA	5
152	-	-	Hepatocerebral	NA	NA	NA	NA	p.Ala86Profs*13	NA	+/0.1	NA	5
153	-	-	Hepatocerebral	NA	NA	NA	NA	p.Ala86Profs*13	NA	+/0.1	NA	5
154	-	-	Hepatocerebral	NA	NA	NA	NA	p.Ala86Profs*13	NA	+/0.1	NA	5

155	-		-	Hepatocerebral	NA	NA	NA	NA	p.Ala86Profs*13	NA	+/0.1	NA	5
156	-		-	Hepatocerebral	NA	NA	NA	NA	p.Ala86Profs*13	NA	+/0.1	NA	5
157	M	0,5	Neonatal	Hepatopathy	NA	↑ / ↑	NA	NA	p.Met1Ile; p.Met1Ile	+	-/12	-	19
158	F	8,0	Infantile	Hepatopathy	NA	↑ / ↑	NA	NA	p.105Arg*; p.Leu250Ser	NA	NA	NA	19
159	M		-	-	NA	NA	NA	NA	p.199Cys*; p.Met1Ile;	NA	NA	NA	19
160	F		-	-	NA	NA	NA	NA	p.Asn276Lysfs*13; p.Met1Ile;	NA	NA	NA	19
161	M		-	-	NA	NA	NA	NA	p.Val137Glu; p.79Gln*	NA	NA	NA	19
162	F		-	-	NA	NA	NA	NA	p.Trp172*; p.Met1Ile;	NA	NA	NA	19
163	M		-	-	NA	NA	NA	NA	p.Arg105*; p.Arg105*	NA	NA	NA	19
164	F		-	-	NA	NA	NA	NA	p.Met1Ile; p.Cys199*	NA	NA	NA	19
165	M		-	-	NA	NA	NA	NA	p.Met1Ile; p.Met1Ile;	NA	NA	NA	19
166	M		-	-	NA	NA	NA	NA	p.Met1Ile; p.Arg105*	NA	NA	NA	19
167	M		-	-	NA	NA	NA	NA	p.Met1Ile; c.143-4312_256+4297del8720	NA	NA	NA	19
168	F		-		NA	NA	NA	NA	p.Met1Ile; p.Met1Ile	NA	NA	NA	19
169	F		-	-	NA	NA	NA	NA	p.Met1Ile; p.Met1Ile	NA	NA	NA	19

170	M		-	-	NA	NA	NA	NA	p.Met1Ile; p.Leu250Ser	NA	NA	NA	19
171	F	6,0	Infantile	Hepatopathy	↑ 1570 U/L	↑51 / ↑83	-	NA	p.Asn46Ser; p.Glu220AspfsTer 23	+	-/0.5	-	UP
172	M		-	-	NA	NA	NA	NA	p.Leu131Thrfs Ter10; p.Ser107Pro	NA	NA	NA	UP
173	F		-	-	NA	NA	NA	NA	p.Ser107Pro;	NA	NA	NA	UP
174	M	1,0	Neonatal	Hepatocerebral	-	↑ / ↑	↑ Ala	NA	p.Glu165Lys	-	+/0.9	MOF, encephalopath y	UP
175	M	0,033	Neonatal	Hepatocerebral	NA	↑ / ↑	NA	-	p.Glu165Lys	-	+/0.4	Liver failure	UP
176	M	6,0	Infantile	Hepatopathy	-	NA / ↑165	↑ Met	NA	p.Glu211Gly; p.Leu266Arg	-	+/1.8	Meningococcu s	UP
177	F	1,2	Infantile	Hepatopathy	NA	NA / ↑240	↑ Tyr	NA	p.Glu165Lys	-	+/1.1	MOF	UP
178	F	5,0	Infantile	Hepatocerebral	NA	NA / ↑615	↑ Tyr, Phe	NA	p.Glu165Lys	-	+/1.4	Liver failure	UP
179	F	0,5	Neonatal	Hepatocerebral	-	NA / ↑955	-	NA	p.Asp265*	-	+/1	Liver failure	UP
180	M	0,2	Neonatal	Hepatocerebral	NA	NA / ↑785	↑ Tyr, Ala	NA	p.Arg118Cys	-	+/0.4	Liver failure	UP
181	M	0,7	Neonatal	Hepatocerebral	NA	NA / ↑175	↑ Tyr	NA	p.Glu44Lys	-	+/0.2	Liver failure	UP
182	F	3,0	Infantile	Hepatomyo cerebral	↑ 500 U/L	↑ 530/ ↑ 880	-	-	p.Tyr149_Lys236 del88	-	+/0.6	Liver failure	UP
183	F	12,0	Infantile	Myopathy	-	- / -	NA	+	p.Asn154Lys; p.His226Arg	-	-/60	-	UP
184	F	0,033	Neonatal	Hepatomyo cerebral	↑ 243 U/L	NA / ↑158	↑ Tyr	NA	p.Glu165Val; c.591+1G>A	-	-/0.3	-	UP
185	F	9,0	Infantile	Hepatomyo cerebral	↑ 13000 U/L	↑111 / ↑59	NA	NA	p.Asn46Ser; p.Arg202TyrfsX12	+	-/26	-	UP

186	M	0,033	Neonatal	Hepatomyo cerebral	NA	↑88 / ↑1114	↑ Tyr	NA	p.Gln79*; p.Gln79*	-	-/0.5	-	UP
187	M	0,033	Neonatal	Hepatopathy	NA	- / ↑150	-	NA	c.143-307_170del; p.Arg118Leu	-	+/0.2	Liver failure	UP
188	M	3,0	Infantile	Hepatocerebral	NA	NA	NA	NA	p.Glu44Lys	-	+/0.8	Liver failure	UP
189	F	1,0	Neonatal	Hepatocerebral	-	↑277 / ↑634	↑ Ala	NA	p.Ser52Phe; p.Asp255Tyr	-	+/1	Liver failure	UP
190	F	0,1	Neonatal	Hepatocerebral	↑ 400 U/L	↑50 / ↑79	↑ Ala	NA	c.300+1G>A	-	+/0.2	Liver failure, GI bleeding	UP
191	F	0,033	Neonatal	Hepatopathy	↑ 390 U/L	↑53 / ↑226	NA	NA	c.300+1G>A	-	+/0.1	Liver failure, GI bleeding	UP
192	M	0,1	Neonatal	Hepatopathy	-	↑57 / ↑140	NA	NA	c.300+1G>A	-	+/0.1	Liver failure	UP
193	M	0,033	Neonatal	Hepatomyo cerebral	↑ 489 U/L	↑190 / ↑269	↑ Ala	NA	c.300+1G>A	-	+/0.3	Liver failure	UP
194	F	0,033	Neonatal	Hepatocerebral	NA	↑221 / ↑51	↑ Tyr	-	p.Glu44Lys;	-	+/0.1	Liver failure	UP
195	M	0,033	Neonatal	Hepatomyo cerebral	-	↑844 / ↑536	-	-	p.Arg105*;	-	+/0.2	Encephalopath y	UP
196	F	3,0	Infantile	Hepatocerebral	NA	↑169 / ↑71	-	-	p.Ala48Glyfs43; p.Gln221Pro	+	+/5.3	Encephalopath y	UP
197	F	0,1	Neonatal	Hepatomyo cerebral	↑ 1022 U/L	↑85 / ↑130	↑ Tyr, Met, Ala, Phe, Gln	NA	p.Ala86Profs*13	-	+/0.1	Liver failure	UP
198	F	2,0	Infantile	Hepatocerebral	NA	NA	NA	NA	p.Ala86Profs*13	NA	NA	NA	32
199	F	6,0	Infantile	Hepatocerebral	NA	NA	NA	NA	p.Phe256*	NA	NA	NA	32
200	F	0,5	Neonatal	Hepatocerebral	NA	NA	↑ Tyr	NA	p.Gln79*	NA	+/1	Liver failure, GI bleeding	26
201	M	2,0	Infantile	Hepatocerebral	NA	NA	↑ Tyr	NA	p.Gln79*	NA	+/0.3	Shock	26

202	F	0,3	Neonatal	Hepatocerebral	NA	- / -	↑ Tyr	NA	p.Gln79*	NA	+/0.2	Metabolic acidosis	26

Cardinal features obtained from the patients' cohort (n=202) were reported. CK: creatin kinase, GI: gastrointestinal, NA: not applicable, MOF: multi-organ failure, UP: updated.

Supplementary Table 2: Clinical manifestations of dGk deficient patients according to the age of onset.

		Neonatal	Infantile	Paediatric	Adult
LIVER	Ascites	23.5%	12.2%	0.0%	0.0%
	Cholestasis	70.6%	57.1%	0.0%	13.3%
	Elevated transaminases	77.6%	83.7%	0.0%	26.7%
	Hepatomegaly	49.4%	59.2%	50.0%	0.0%
	Jaundice	74.1%	61.2%	0.0%	0.0%
CNS	Hypotonia	66.7%	48.0%	0.0%	0.0%
	Lethargy	14.3%	4.0%	0.0%	0.0%
	Nystagmus	29.8%	30.0%	0.0%	0.0%
	Psychomotor delay	32.1%	30.0%	0.0%	0.0%
MUSCLE	Dysphagia	4.9%	0.0%	0.0%	53.3%
	Dysphonia	2.4%	0.0%	25.0%	33.3%
	Myalgia	0.0%	4.2%	25.0%	60.0%
	Ophthalmoplegia	0.0%	2.1%	25.0%	80.0%
	Ptosis	6.1%	4.2%	25.0%	93.3%
	Weakness/fatigue	14.6%	12.5%	50.0%	86.7%

Percentage of sign and symptoms of patients stratified according to disease age of onset: neonatal onset (≤ 1 month), infantile (>1 month and ≤ 1 year), pediatric (>1 year and ≤18 years) and adult (>18 years). Data were available for 85 neonatal patients, 49 infantile patients, 4 paediatric patients and 15 adult patients.

Supplementary Table 3 Patients classification based on their clinical symptoms at-onset and during the disease course.

Clinical Form	%	Age at onset		Cardinal Symptoms: CNS		Cardinal Symptoms: Liver		Cardinal Symptoms: Muscle		Additional Symptoms	
**Hepatocerebral**	110 (58.8%)	Neonatal	54/83 (65.1%)	Hypotonia	71 (64.5%)	Ascites	17 (15.5%)			Failure to thrive	40 (36.4%)
		Infantile	29/83 (34.9%)	Lethargy	7 (6.4%)	Cholestasis	61 (55.5%)			Feeding difficulties	33 (30.0%)
		Paediatric	-	Nystagmus	51 (46.4%)	Elevated Transaminases	67 (60.9%)			Hypothermia	10 (9.1%)
		Adult	-	Psychomotor Delay	31 (28.2%)	Hepatomegaly	51 (46.4%)			Hypoglycaemia	67 (60.9%)
						Jaundice	64 (58.2%)			Metabolic acidosis	75 (68.2%)
										Renal involvement	10 (9.1%)
**Hepatomyocerebral**	18 (9.6%)	Neonatal	14/18 (77.8%)	Hypotonia	16 (88.9%)	Ascites	4 (22.2%)	Dysphagia	4 (22.2%)	Failure to thrive	1 (5.6%)
		Infantile	4/18 (22.2%)	Lethargy	7 (38.9%)	Cholestasis	10 (55.6%)	Dysphonia	2 (11.1%)	Feeding difficulties	11 (61.1%)
		Paediatric	-	Nystagmus	8 (44.4%)	Elevated Transaminases	13 (72.2%)	Muscle pain	1 (5.6%)	Hypothermia	3 (16.7%)
		Adult	-	Psychomotor Delay	11 (61.1%)	Hepatomegaly	7 (38.9%)	Ptosis	6 (33.3%)	Hypoglycaemia	14 (77.8%)
						Jaundice	12 (66.7%)	Ophthalmoplegia	-	Metabolic acidosis	16 (88.9%)
								Weakness	15 (83.3%)	Renal involvement	2 (11.1%)
**Isolated hepatopathy**	41 (21.9%)	Neonatal	20/39, (51.3%)			Ascites	5 (12.2%)	Dysphagia	-	Failure to thrive	1 (2.4%)
		Infantile	17/39, 43.6%)			Cholestasis	19 (46.3%)	Dysphonia	-	Feeding difficulties	3 (7.3%)
		Paediatric	2/39, 5.1%)			Elevated Transaminases	32 (78.0%)	Muscle pain	1 (2.4%)	Hypothermia	3 (7.3%)
		Adult	-			Hepatomegaly	15 (36.6%)	Ptosis	-	Hypoglycaemia	19 (46.3%)
						Jaundice	18 (43.9%)	Ophthalmoplegia	-	Metabolic acidosis	14 (34.1%)
								Weakness	2 (4.9%)	Renal involvement	3 (7.3%)

		Neonatal	-		Ascites	-	Dysphagia	8 (44.4%)	Failure to thrive	-
		Infantile	1/18 (5.6%)		Cholestasis	2 (11.1%)	Dysphonia	6 (33.3%)	Feeding difficulties	2 (11.1%)
		Paediatric	2/18 (11.1%)		Elevated Transaminases	4 (22.2%)	Muscle pain	10 (55.6%)	Hypothermia	-
**Isolated myopathy**	18 (9.6%)	Adult	15/18 (83.3%)		Hepatomegaly	-	Ptosis	16 (88.9%)	Hypoglycaemia	-
					Jaundice	-	Ophthalmoplegia	14 (77.8%)	Metabolic acidosis	1 (5.6%)
							Weakness	16 (88.9%)	Renal involvement	-
							Motor delay	1 (5.6%)		

Data obtained from 187 patients (15 N/A) were classified based on their clinical symptoms at-onset and during the disease course into the following four clinical subgroups: (i) hepatocerebral: patients showed signs/symptoms of the liver (jaundice, cholestasis, hepatomegaly, ascites, elevated transaminases, liver failure) and brain (central hypotonia, psychomotor delay, nystagmus, lethargy) involvement; (ii) hepatomyocerebral: in case of liver, brain and muscle involvement (weakness/fatigue, ptosis, ophthalmoplegia, myalgia, dysphonia, dysphagia); (iii) hepatic: isolated involvement of the liver; (iv) myopathic: isolated involvement of the muscle.
Age at onset was not available in 27 patients with hepatocerebral form and 2 patients with isolated hepatopathy.
Abbreviations: (-): No patient presented the specifical signs or symptoms; CNS: Central Nervous System. Each value is expressed as: value and (percentage).
